# Biophysical characterization of the homodimers of HomA and HomB, outer membrane proteins of *Helicobacter pylori*

**DOI:** 10.1038/s41598-021-04039-4

**Published:** 2021-12-28

**Authors:** Anubhav Tamrakar, Rahul Singh, Amit Kumar, Ravindra D. Makde, Ashish, Prashant Kodgire

**Affiliations:** 1 Discipline of Biosciences and Biomedical Engineering, Indian Institute of Technology Indore, Indore, Madhya Pradesh 453 552 India; 2 High Pressure and Synchrotron Radiation Physics Division, Bhabha Atomic Research Center, Trombay, Mumbai, India; 3 Protein Science and Engineering Division, CSIR-Institute of Microbial Technology, Chandigarh, India

**Keywords:** Chemical biology, Proteins, Membrane proteins

## Abstract

*Helicobacter pylori* is a Gram-negative bacterium that causes chronic inflammations in the stomach area and is involved in ulcers, which can develop into gastric malignancies. *H. pylori* attaches and colonizes to the human epithelium using some of their outer membrane proteins (OMPs). HomB and HomA are the most studied OMPs from *H. pylori* as they play a crucial role in adherence, hyper biofilm formation, antibiotic resistance and are also associated with severe gastric malignancies. The role of HomA and HomB in pathogenesis concerning their structure and function has not been evaluated yet. In the present study, we explored the structural aspect of HomA and HomB proteins using various computational, biophysical and small-angle X-ray scattering (SAXS) techniques. Interestingly, the in-silico analysis revealed that HomA/B consists of 8 discontinuous N and C terminal β-strands forming a small β-barrel, along with a large surface-exposed globular domain. Further, biophysical experiments suggested that HomA and HomB are dimeric and most likely the cysteine residues present on surface-exposed loops participate in protein–protein interactions. Our study provides essential structural information of unexplored proteins of the Hom family that can help in a better understanding of *H. pylori* pathogenesis.

## Introduction

Gastric cancer is the second most common cause of cancer-associated deaths^1^. Chronic gastritis is the major cause of human gastric cancer caused by the Gram-negative bacterium *Helicobacter pylori*. The International Agency for Research on Cancer (IARC) declared *H. pylori* as a type-1 carcinogen for gastric cancer, which is mediated via chronic gastric inflammation*. H. pylori* is estimated to infect 50% of the world’s population^2–4^. The long-term persistence of *H. pylori* can stimulate a severe immune response that can damage the mucosal lining. Chronic inflammation due to *H. pylori* infection makes it a potentiate agent of acute and chronic gastritis, peptic ulcer disease (PUD), and two forms of cancers, namely, mucosa-associated lymphoid tissue (MALT) lymphoma and gastric adenocarcinoma^5–8^.

Outer membrane proteins (OMPs) of *H. pylori* play a crucial role in the host–pathogen interaction, virulence and pathogenesis. Upon interaction with the host immune cells, OMPs stimulate the production of pro-inflammatory molecules that may result in an uncontrolled inflammation leading to the transformation of a normal cell to a cancerous cell. Hom (Helicobacter outer membrane) family of OMPs in *H. pylori* consists of four members (HomA, B, C and D). In the *H. pylori* genome *jhp*0870 open-reading frame (ORF) that codes for HomB outer membrane protein is associated with many stomach diseases and is a novel co-marker for peptic ulcer disease (PUD)^9,10^. *homB* gene is 90% similar to another OMP of the *H. pylori*, which is the *jhp*0649 ORF *homA,* with differences between the 300 bp middle region of these ORFs^11^. The Hom OMP family consists of four members, of which *homB* is the most studied, and evaluated for its prevalence in various peptic diseases. Oleastro et al. investigated pathogenesis and immunological response caused by HomB protein in clinical patients and 190 *H. pylori* strain isolated from patients with peptic ulcer disease (PUD) or gastritis were evaluated for the clinical importance of *homB*. HomB protein contributes to the colonization and persistence of *H. pylori*, and the presence of *homB* genes affects the number of bacteria adhering to the host cells. Additionally, HomB is also associated with the secretion of the proinflammatory cytokine interleukin-8 (IL-8)^12^. With the agreement of these studies, *hom*B can be considered as a virulence marker of *H. pylori* virulent strains. However, it is not clear how HomB contributes to ulcer or gastric cancer, and the exact molecular mechanism is not been explored yet.

Outer membrane proteins (OMPs) are distinctive features of Gram-negative bacteria. Most of the OMPs from Gram-negative bacteria form β-barrel, such as OmpA, PhoE, LamB, OmpF, OmpC, FepA, and FhuA from *E. coli*. OMPs are composed of multiple β-sheets arranged in an anti-parallel fashion and thus form a β-barrel. For example, OmpX, from *E. coli*^13^ and PagP from *Salmonella typhimurium* possess 8 β-stranded structures^14^. Additionally, large β-barrel proteins also have been characterized, such as 26 β-strands LptD from *Shigella flexneri*^15^, 24-stranded β-barrel PapC from *E. coli*^16^.

These β-barrel proteins contain the transmembrane domains which interact with the host cell receptors. Additionally, these proteins form a porin-like structure and act as efflux pumps, and are involved in the transport of metabolites, such as BtuB, OprM^17,18^. Interestingly, these transmembrane β-barrels can exist as monomers or oligomers, for example, OmpF from *Salmonella typhimurium* forms a trimer of β-barrel^19^. Interestingly, TolC trimer from *E. coli* forms a single β-barrel^20^. The β-barrel proteins have the lipid facing hydrophobic and protein facing hydrophilic residues which are arranged alternatively in the transmembrane, enabling the formation of a pore-like structure and thus making them highly stable. Extensive inter-strand hydrogen bonding with the non-polar lipid bilayer and the existence of aromatic residues at the water-bilayer interface anchor them in the lipid bilayer and thus result in highly stable transmembrane β-barrels, which do not unfold easily^21–23^.

Despite the clinical significance of HomA and HomB, their structural characteristics have not been studied in detail. Here, we performed bioinformatics analysis for HomA as well as HomB and observed that these OMPs likely form a small β-barrel, along with a large surface-exposed globular domain. Later, we expressed and purified HomA and HomB, and further used them for their biophysical characterization, using various spectroscopic as well as chromatographic techniques. We also used various detergents and lipids to analyze the structural properties of these OMPs in different conditions. Additionally, SAXS was performed to reveal the three-dimensional shape of these OMPs. These structural characteristics are unique to the *H. pylori* Vf class of autotransporter which are recently reported for Hop family protein^24^ and the least explored for their functional properties.

## Results

### In-silico analysis of HomA and HomB sequence

**Figure 1:**
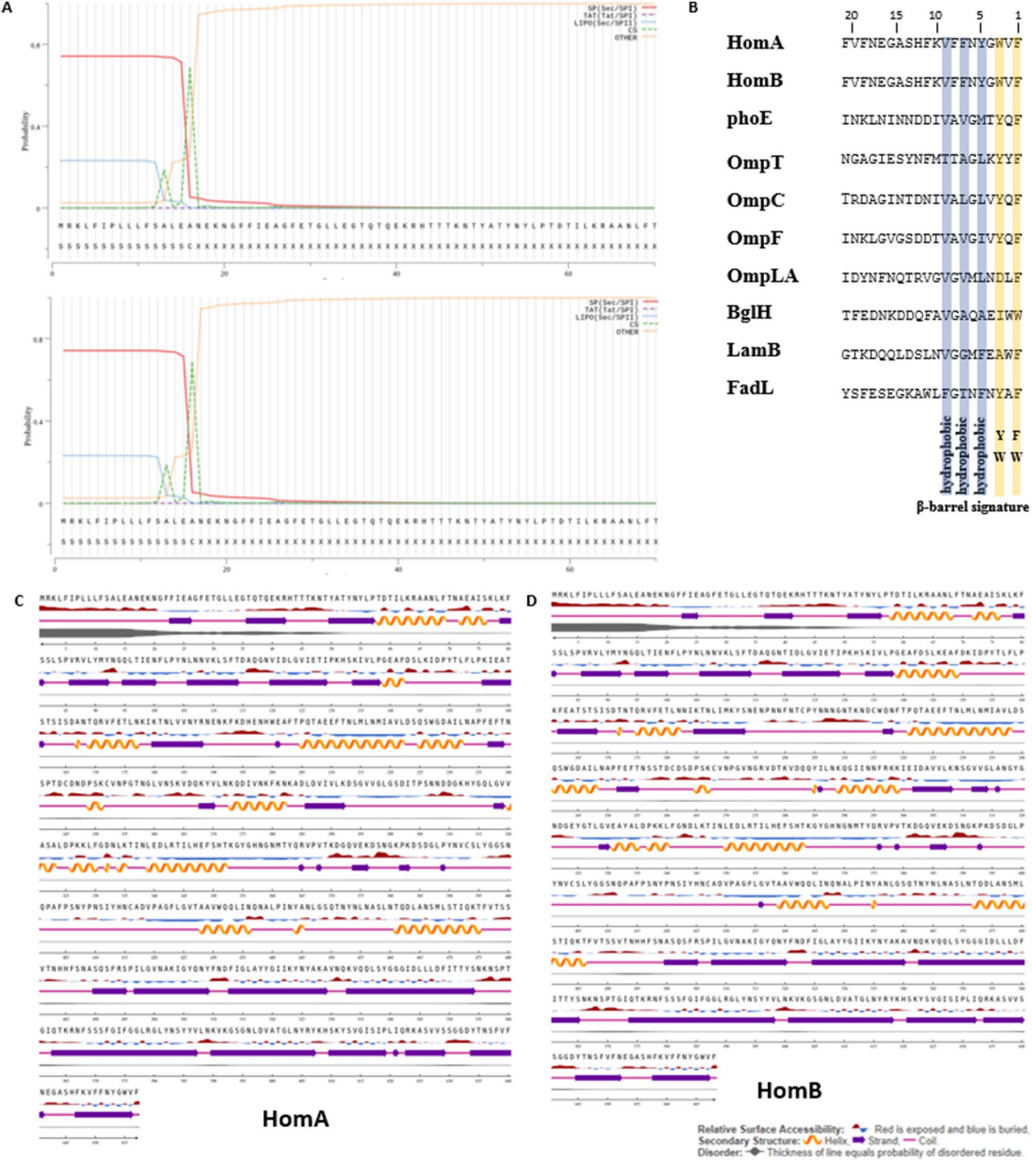
In silico studies for HomA and HomB. (**A**) N-terminal outer membrane protein signal sequences prediction of HomA and HomB using SignalP-5.0 (http://www.cbs.dtu.dk/services/SignalP/)^25^. (**B**) C-terminal β-barrel signature sequence comparison among reported bacterial β-barrel outer membrane proteins with HomA and HomB. (**C**,**D**) Secondary structure contents prediction of HomA and HomB, respectively, using NetSurfP-2.0 (http://www.cbs.dtu.dk/services/NetSurfP/)^27^.

Structural aspects of outer membrane protein HomA and HomB from *H. pylori* in bacterial pathogenesis have not been extensively explored. Being membrane proteins, HomA and HomB are expected to contain a signal peptide that will enable its insertion in the outer membrane of bacteria. Thus, to identify the presence of a signal peptide for HomA and HomB, we analyzed amino acid sequences using the tool SignalP-5.0^25^ and observed that both HomA and HomB possess a 16 amino acid N-terminal signal peptide (Fig. 1A), like other Gram-negative bacteria. Typically, outer membrane proteins from Gram-negative bacteria are synthesized and recognized by the BAM assembly complex via C-terminal consensus sequence X-Z-X-Z-X-Z-Tyr-Z-Phe/Trp (where X is any hydrophobic and Z is any amino acid)^26^. To confirm the presence of such a signal at the C-terminal, we performed a sequence alignment with the majority of β-barrel proteins from *E. coli* and observed that HomA and HomB also show identical C-terminal consensus signature sequences for β-barrel OMPs (Fig. 1B).

Further, to determine the secondary structure for HomA and HomB, we used the tool NetSurfP-2.0^27^ and observed that HomA and HomB are likely made up of mixed α helices, β-sheets, and random coils topology, where 22% α-helices, 34% β-sheets and 44% random coils contribute for HomA, and 18.5% α-helices, 38.5% β-sheets and 43% random coils contribute for HomB. Remarkably, N-terminal shows high scale disordered residues, which might be inside or outside the membrane as NetsurfP 2.0 predicts only the secondary structure content and not the location topology (Fig. 1C,D).

**Figure 2:**
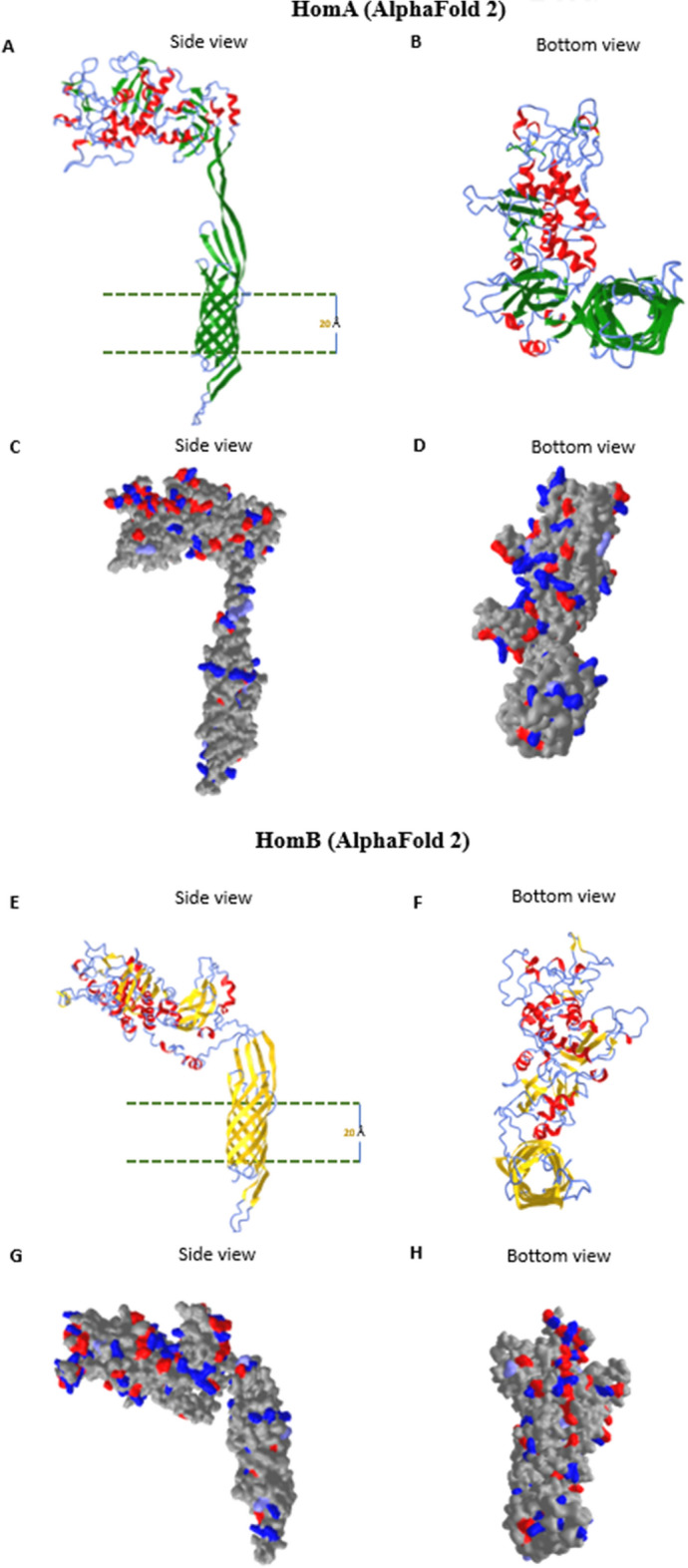
Alphafold 2 3D structure prediction of HomA and HomB. (**A–H**) 3D structure predictions of HomA and HomB (without signal peptide) using Alphafold2^28^, side and bottom view for HomA from left to right (**A**,**B)** β-sheets (green), α-helices (red) and coils (blue); side and bottom view for HomB from left to right (**E**,**F**) β-sheets (yellow), α-helices (red) and coils (blue). (**C**,**D**) molecular surface view of charged residues of HomA and (**G**,**H**) for HomB.

As both HomA and HomB predicted to have a significant number of β-sheets, to know whether these are transmembrane β-strands of outer membrane proteins, we predicted the three dimensional (3D) structure of HomA and HomB to know the organization of these proteins inside the outer membrane of the bacteria. Here, we employed AlphaFold 2^28^ and I-TASSER^29^ for the prediction of the 3D structure model and observed that both HomA and HomB form β-barrel structures (Fig. 2A–H). AlphaFold 2 predicted a small β-barrel domain that is rich in β-sheets, along with a large surface-exposed globular domain which consists of α-helix, β-sheets and random coils. Based on the AlphaFold 2 predictions for HomA and HomB, secondary structure contents are 20% α-helix, 43% β-sheets, 37% random coil, and 16% α-helix, 38% β-sheets, 46% random coil, respectively. These predicted models contain 27 β-strands for HomA, and 28 β-strands for HomB, in total. Interestingly, the small barrels for HomA and HomB were made up of 8 β-strands, where N-terminal S1 β-strand is a part of β-barrel which extend to a surface-exposed globular domain and S19 β-strand comes back to be a part of β-barrel. Likely to HomA, N-terminal S1 β-strand of HomB is a part of β-barrel extends to the surface-exposed globular domain and S20 β-strand comes back to be a part of β-barrel (Supplementary Fig. S3A,B). We also observed hydrophobic belt around the membrane-embedded portions (20 Å calculated) and predominance of charged residues in both outer as well as the inner portion of the barrel for binding to the lipopolysaccharides molecules, as shown in the molecular surface representation in HomA (Fig. 2C,D) and HomB (Fig. 2G,H) models. These in-silico studies suggest that HomA and HomB form a small β-barrel outer membrane protein consisting of 8 β-strands, along with a large surface-exposed globular domain.

### Cloning, expression and purification of HomA and HomB

*homA* and *homB* genes were cloned in *E. coli* to obtain purified HomA and HomB proteins, which were expressed with a C-terminal His-tag. *homA* and *homB* genes were PCR amplified from the genomic DNA of *H. pylori* strain J99 with primers (Supplementary Table S1) and cloned into pBluescript. Based on the restriction pattern, we identified unique clones for HomA and HomB and later confirmed them by DNA sequencing. Subsequently, both the HomA and HomB genes were cloned into pET43 at *Nde*I and *Xho*I sites, respectively, to give pHomA and pHomB (Supplementary Fig. S1A). Cloning of *homA*/*homB* in pET43 was confirmed by restriction analysis followed by DNA sequencing. The resultant recombinants were transformed into *E. coli* Rosetta (DE3) cells and induced with 0.1 mM isopropyl-b-d-1-thiogalactopyranoside (IPTG). Induced cells were harvested, washed, and resuspended in 500 µl of buffer containing 25 mM Tris–Cl, pH 7.4. Cell lysates were prepared and separated as supernatant and pellet, and supernatant samples were analyzed on 8% SDS-PAGE gels to evaluate the amount of protein present in the soluble form. A high level of protein expression of HomA and HomB was observed in the supernatant of induced cells but was absent in the uninduced cells (Fig. S1B and C; lanes 2 and 3).

The cloning of *homA* and *homB* was performed in such a way that the recombinant protein codes for a C-terminal His-tag (Supplementary Fig. S1A), for affinity purification. HomA and HomB were purified using a Ni–NTA column using the Akta system (GE healthcare). The input sample was prepared in the equilibration buffer (50 mM Tris–Cl buffer, pH 8.0, 300 mM NaCl, and 10 mM imidazole), and loaded on the pre-equilibrated column at a flow rate of 1 ml/min. Subsequently, the column was washed with a wash buffer comprising 50 mM Tris–Cl, pH 8.0, 300 mM NaCl, with 50 mM imidazole. The sample was eluted using elution buffer comprising 50 mM Tris–Cl, pH 8.0, 300 mM NaCl, with 250 mM imidazole. The samples were collected for analysis by SDS-PAGE. Fig. S1B and C show that the HomA and HomB proteins were able to bind to the Ni–NTA resin, as most of the protein was absent in the flow-through (Fig. S1B and C, lane 4). The size of purified proteins was observed near to 75 kDa on 8% SDS-PAGE for both HomA and HomB. We were able to purify more than 95% pure HomA and HomB proteins (Supplementary Fig. S1B and C, lane 5).

**Figure 3:**
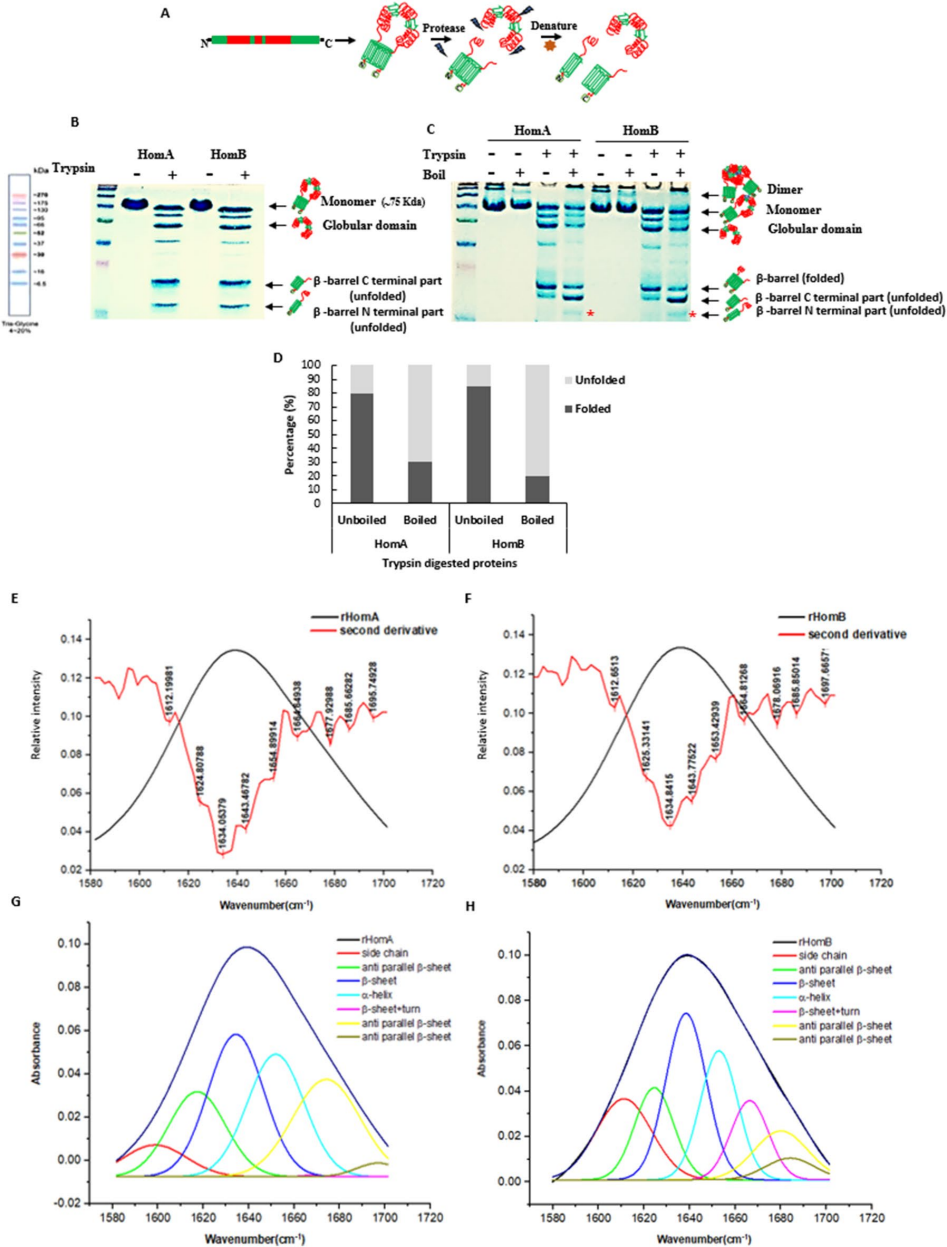
Trypsin digestion analysis and ATR-FTIR spectroscopy of HomA and HomB. (**A**) pictorial representation of HomA and HomB protease digestion of scheme. (**B**) trypsin digested proteins run onto denaturing SDS-PAGE. (**C**) same protein sample run onto the semi-native gel with and without boiling. (**D**) percentage of folded and unfolded fractions observed on semi-native PAGE. The red asterisk (*) denotes the N-terminal part that comes out upon unfolding. (**E–H**) Secondary structure analysis using curve fitting of the amide I region (1600–1700 cm^−1^) ATR-FTIR spectroscopy. (**E**,**F**) FTIR absorbance spectra of amide 1 of HomA and HomB, respectively, with their second derivatives. (**G**,**H**) Deconvolution of amide 1 spectra of HomA and HomB, respectively, for secondary structure content analysis. The spectral range of 1600–1700 cm^−1^ was decomposed using Origin 8.0 software. [Full gel image for (**B**) and (**C**) are in Supplementary Fig. S8].

To investigate the reliability of predicted 3D models of HomA and HomB, purified proteins were treated with protease. Trypsin digestion was carried out for one hour at 37 °C followed by further analysis via either SDS reducing PAGE or semi-native PAGE (Fig. 3A–C). A cartoon representation depicts the possible outcomes of trypsin digestion for HomA and HomB (Fig. 3A). Incidentally, trypsin has several cleavable sites on HomA and HomB protein sequence, nevertheless, typically β-barrel domains show resistance to protease digestion. As expected from the predicted models (Fig. 2), the surface-exposed globular domain part corresponds to 40–45 kDa in addition to a 30–35 kDa band of small β-barrel on the gel. Most likely, the N-terminal (16–18 kDa) and C-terminal (22–25 kDa) regions, for HomA and HomB which are rich in β-sheets, come together to form a β-barrel. Interestingly, we observed 3 dominant bands of approximately 45 kDa, 25 kDa and 18 kDa both for HomA and HomB (Fig. 3B). Since β-barrel is likely to be made up of N and C terminals, on a reducing gel two separate bands were observed of the size of around 25 kDa and 18 kDa (Fig. 3B). To check whether these two bands are part of β-barrel, the same trypsin digested protein samples were run on a semi-native PAGE. Interestingly, after the denaturation of these samples, we observed two separate bands of the β-barrel domain. The single band in the unboiled samples of HomA and HomB suggests that 80–85% protein is in folded conformation, whereas the boiled samples show only 25–30% fraction in the folded conformation (Fig. 3C,D). The additional denatured protein band of the β-barrel domain was observed at around 16–18 kDa (Fig. 3C, lane 5 and 9) which is also present in SDS reducing PAGE (Fig. 3B), however, this band was not present in the unboiled protein samples on the semi-native gel (Fig. 3C, lane 4 and 8). Typically, β-barrel proteins show migration differences in boiled and unboiled protein samples during PAGE analysis. Folded β-barrel shows faster migration, whereas unfolded fraction shows slower migration on PAGE. In the case of HomA and HomB, β-barrel is most likely composed of N and C terminal β-strands and upon unfolding that become two separate parts and these migrate as lower size bands. These observations support the in silico predicted model of HomA and HomB (Fig. 2).

We also tested the heat modifiability of undigested purified HomA and HomB proteins in the presence of different detergents and lipids (Supplementary Fig. S1D,E). We did not observe a significant migration difference for purified HomA, however, upon incubation with detergents LDAO, TWEEN 20, CHAPS (4X CMC) and lipids DOPC and DMPC (1 mM), a slight migration difference was observed (Supplementary Fig. S1D). Similarly, in the case of purified HomB and HomB incubated with detergents LDAO, TWEEN 20 and lipid DOPC show a slight migration difference, whereas DMPC and CHAPS show no heat modifiability (Supplementary Fig. S1E). Since HomA and HomB are likely to contain a small β-barrel with a large globular domain composed of α-helix, β-sheets and random coils and this is possibly the main cause of masking the heat modifiability of these proteins.

### HomA and HomB are rich in β-sheet and likely form barrel topology

The IR spectra of HomA and HomB proteins were collected to determine and confirm the predicted secondary structures. The secondary structure information of a protein’s IR spectrum lays under the Amide I region, which is made up of several overlapping spectra that are composed of secondary structure elements, such as β-sheet, α-helix, turns, loops and some of them also consist of the side chain absorbance. Here, the Amide I band in the range 1700–1600 cm^−1^ was plotted with its secondary derivatives. To analyse the secondary structure content of Amide I, we decomposed it to see the individual component spectra position. The second derivative calculation is the easiest and the most common method among a few others, which was employed for data analysis. We observed that HomA and HomB both contain high anti-parallel β-sheet as the peaks were observed at 1634 cm^−1^, 1685 cm^−1^, 1695 cm^−1^_,_ and 1634 cm^−1^, 1685 cm^−1^, 1697 cm^−1^, respectively. However, both proteins also show a peak at 1654 cm^−1^ for α-helix with a disordered structure peak at 1643 cm^−1^ (Fig. 3E,H, Supplementary Table S2).

**Figure 4:**
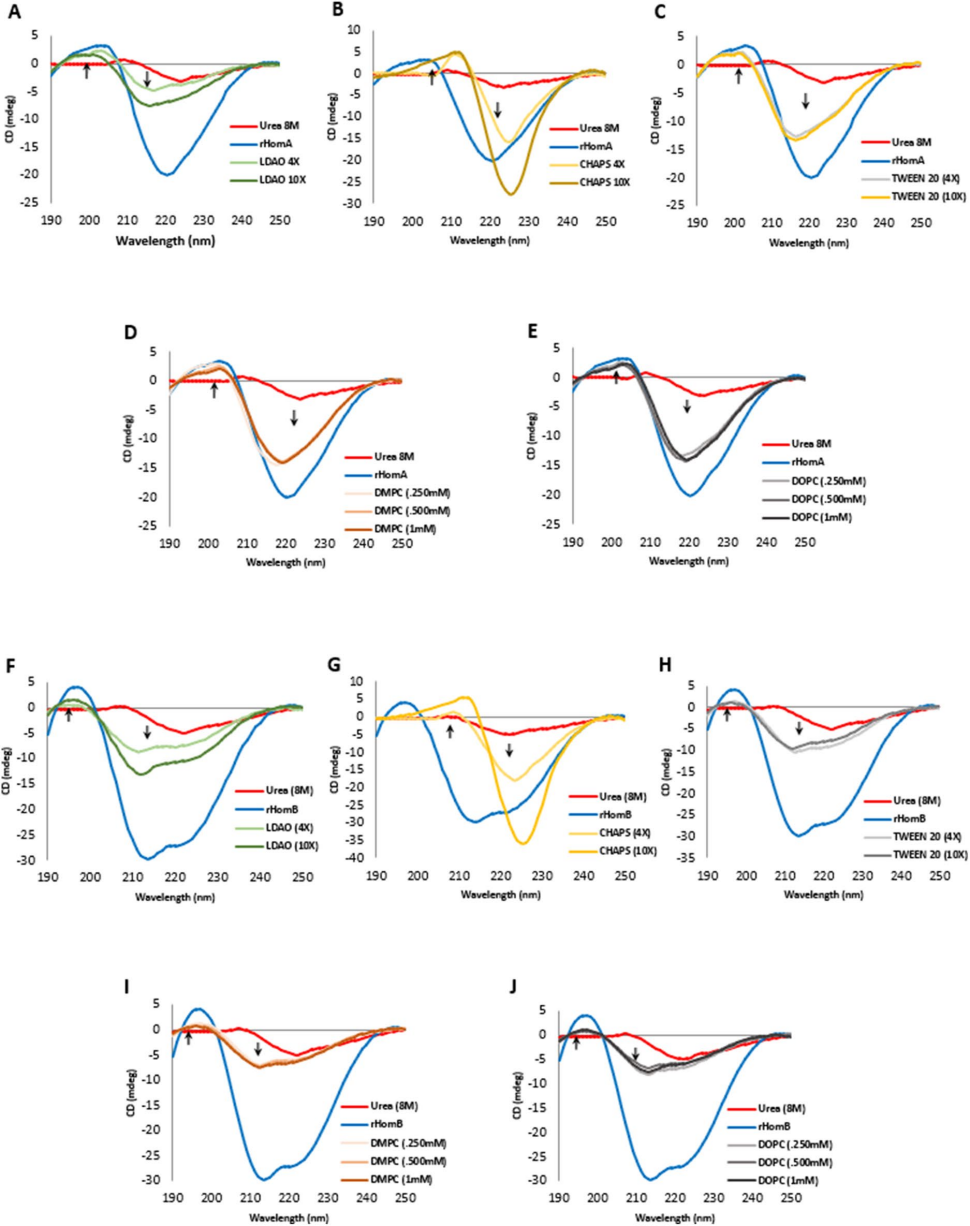
Secondary structure Analysis using circular dichroism. (**A**–**C**) CD spectra for HomA, 40-fold dilution to buffer containing detergent ×4 and ×10 concentration of CMC, LDAO, CHAPS, and TWEEN 20, respectively. (**D**,**E**) CD spectra for HomA, 40-fold dilution to a buffer containing 0.25 mM, 0.5 mM, and 1 mM DMPC and DOPC lipids, respectively. (**F**–**H**) CD spectra for HomB, 40-fold dilution to buffer containing detergent ×4 and ×10 concentration of CMC, LDAO, CHAPS, and TWEEN 20, respectively. (**I**,**J**) CD spectra for HomB, 40-fold dilution to a buffer containing 0.25 mM, 0.5 mM, and 1 mM DMPC and DOPC lipids, respectively.

We performed circular dichroism (CD) experiments to know the secondary structure content of HomA and HomB. Despite sharing more than 92% identity (Supplementary Fig. S2), CD spectra of HomA and HomB are unique. Soluble HomA protein CD spectra show a broad negative peak at around 220 nm and a positive peak at 203 nm, whereas, soluble HomB protein CD spectra show two negative peaks at 214 nm and 221 nm, respectively, and a positive peak at 197 nm (Fig. 4). Analysis of the CD spectra revealed that the proportion of α-helix (10.5%), β-sheet (39.6%) and Turn (11.5%) for HomA, and α-helix (20%), β-sheet (25.6%), and Turn (14.4%) for HomB.

Membrane and detergent environments tend to be amphipathic, and therefore they have different physical properties than aqueous solutions, producing different spectral characteristics for proteins embedded in them. As urea denatured OMPs can refold in micelles of detergents and lipids, we performed CD experiments to study the refolding properties of HomA and HomB. Supplementary Table S3 summarizes deconvoluted data of CD spectra of HomA and HomB in the presence of various lipids and detergents. HomA and HomB show hypsochromic shifts in the presence of LDAO (4× and 10× CMC), DMPC and DOPC (0.25 mM, 0.5 mM, and 1 mM), and TWEEN 20, indicating that incubation of HomA and HomB with these lipids and detergents reduce α-helix content and increases the parallel β-sheet structure. Contrarily, the addition of CHAPS (4× and 10× CMC) to denatured HomA and HomB proteins did not increase the parallel β-sheet structure (Supplementary Table S3).

HomA with CHAPS (4× and 10× CMC) reduces α-helix content and increases the turn structures, HomA with TWEEN 20 and LDAO (4× and 10× CMC) reduces α-helix content and increases the parallel β-sheet structure. While, the addition of lipids, such as DMPC and DOPC (0.25 mM, 0.5 mM, and 1 mM) to HomA, demonstrated reduced α-helix content and increased mixed parallel and anti-parallel β-sheet as well as turns in the CD spectra (Fig. 4A–E, Supplementary Table S3). Likewise, CD spectra analysis of HomB with CHAPS (4× and 10× CMC) demonstrated a reduction in α-helix content and increased turn structures, up to 48%. Similarly, HomB with TWEEN 20 and LDAO (4× and 10× CMC) revealed a reduction in α-helix content and an increase in the parallel and anti-parallel β-sheet structure. Very similar to HomB, the addition of lipids, such as DMPC and DOPC (0.25 mM, 0.5 mM, and 1 mM) to HomA, demonstrated a reduction in α-helix content and an increase in the mixed parallel and anti-parallel β-sheets (Fig. 4F–J, Supplementary Table S3).

### Effect of pH, detergents, and lipids on the topology of HomA and HomB

Incidentally, HomA and HomB both contain 4 tryptophan amino acid residues (Supplementary Fig. S3). The tryptophan residues give fluorescence emission peaks between 300 to 350 nm depending on the polarity of the local environment, and thus can be used to assay predicted topologies. We measured tryptophan fluorescence of recombinant HomA and HomB proteins to study the conformational state of a protein. A fully folded β-barrel protein gives fluorescence emission maximum near to 330 nm range, called blue-shift, as the tryptophan residues present in the folded β-barrel proteins move from an aqueous environment to the hydrophobic environment^30^. We tested the folding pattern of denatured and purified HomA and HomB proteins in various pH buffers, at different urea concentrations, as well as in the presence of various detergents and lipids.

**Figure 5:**
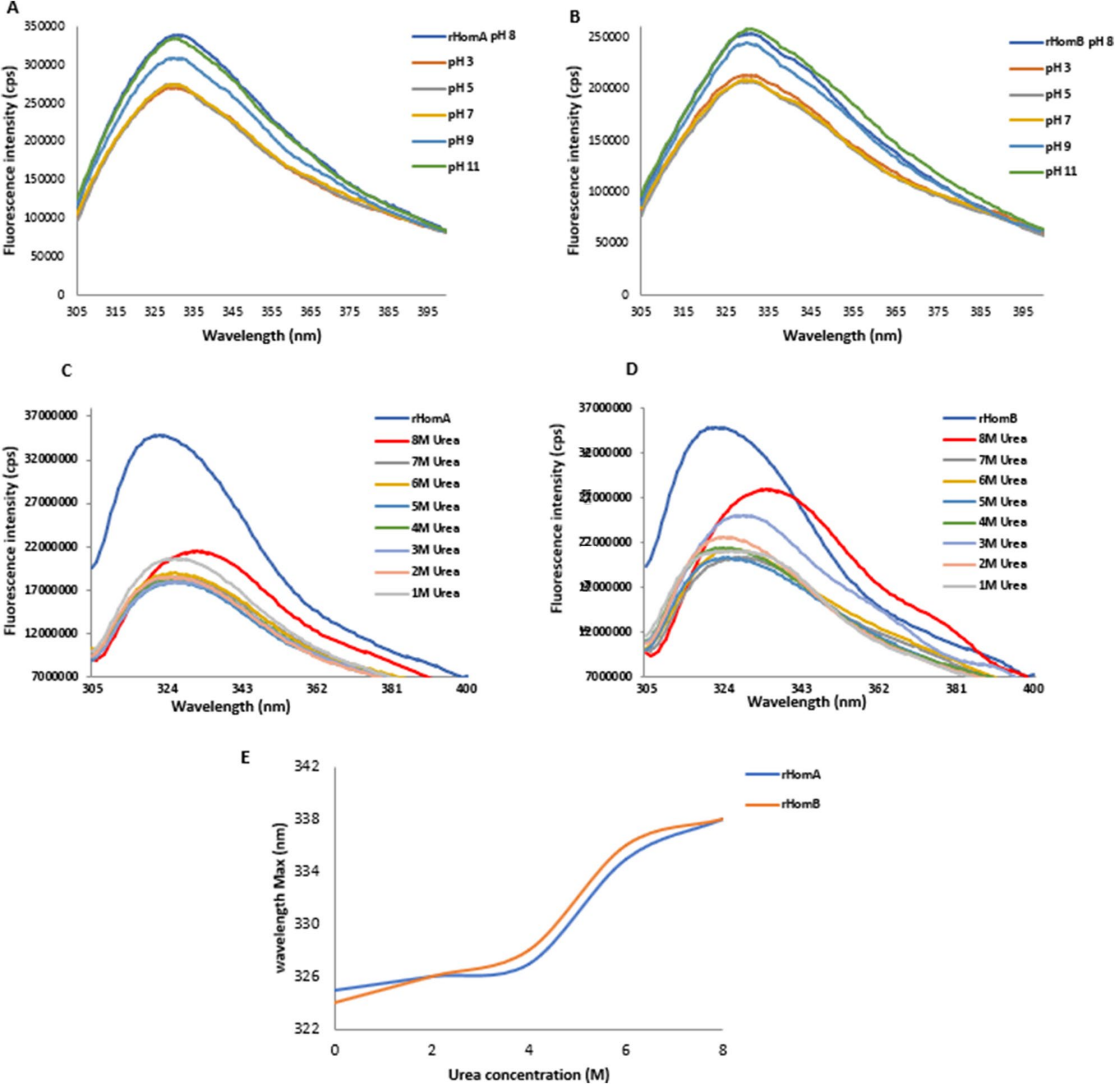
Effect of pH and urea on HomA and HomB. (**A**,**B**) Effect of pH on HomA and HomB Tryptophan fluorescence, respectively. (**C**,**D**) Tryptophan fluorescence spectra of HomA and HomB, respectively, with increasing urea concentration from 1 to 8 M. (**E**) Representation of denaturation kinetics for HomA and HomB, respectively, with increasing concentration of urea.

Initially, we checked the effect of pH on HomA and HomB structures, by incubating these proteins in buffers ranging from pH 3.0 to 11.0. We observed that changes in the pH do not affect the structure of these proteins, and it shows fluorescence maxima at 324 nm for HomA and 323 nm for HomB, respectively (Fig. 5A,B). Subsequently, we studied the denaturation kinetics of HomA and HomB by employing an increasing concentration of urea from 1 to 8 M. We checked the effect of urea on HomA and HomB structures, by incubating these proteins in buffers containing 1 M to 8 M urea, to understand the denaturation pattern. The addition of urea resulted in the redshift (bathochromic shift) from 324 to 338 nm for HomA, and 323 nm to 340 nm for HomB, respectively (Fig. 5C–E). The unfolding of these proteins started at 4 M urea, and a steady decrease in the fluorescence intensity suggests that these proteins are changing their conformations in the presence of increasing concentrations of urea.

**Figure 6:**
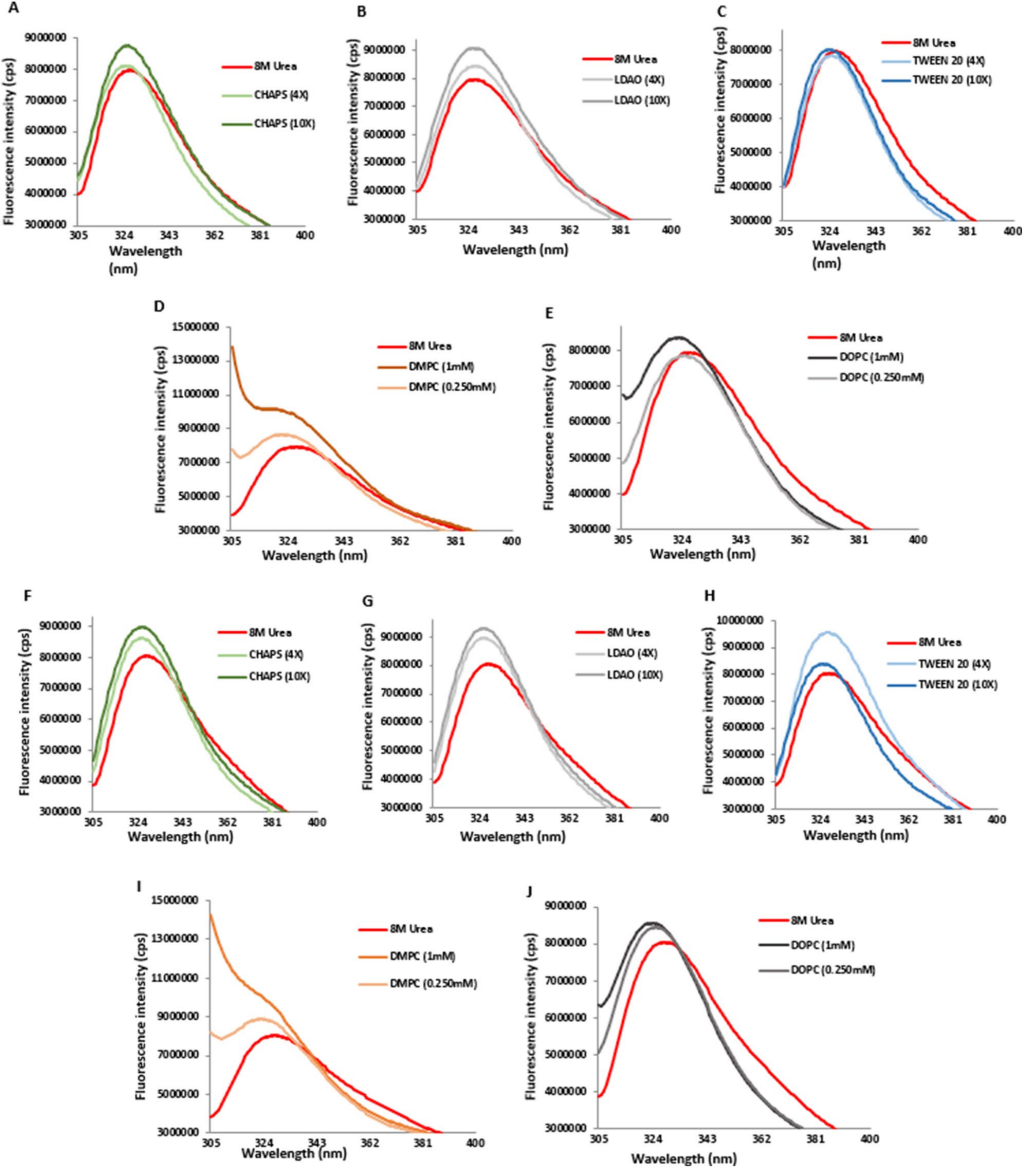
Effect of detergents and lipids on HomA and HomB folding. (**A**–**C**) Tryptophan fluorescence spectra of denatured HomA and 40 folds diluted in buffer containing CHAPS, LDAO, and TWEEN 20 detergents, respectively, ×4 and ×10 concentration of CMC. (**D**,**E**) Tryptophan fluorescence spectra of denatured HomA and 40 folds diluted in buffer containing DMPC and DOPC (0.25 mM and 1 mM), respectively. (**F**–**H**) Tryptophan fluorescence spectra of denatured HomB and 40 folds diluted in buffer containing CHAPS, LDAO, and TWEEN 20 detergents, respectively, ×4 and ×10 concentration of CMC. (**I**,**J**) Tryptophan fluorescence spectra of denatured HomB and 40 folds diluted in buffer containing DMPC and DOPC (0.25 mM and 1 mM), respectively.

To understand the refolding pattern from its denatured unfolded states in the presence of urea, we performed a 40-fold dilution of proteins using a buffer containing different concentrations of detergents and lipids, thus diluting urea concentration from 8 M to less than 300 mM. The unfolded HomA protein in 8 M urea has an emission maximum at 338 nm, whereas the folded protein shows an emission maximum at 324–325 nm for CHAPS (4×) and (10×), 327–328 nm for LDAO (4×) and (10×), 325–326 nm for TWEEN 20 (4×) and (10×), 324 nm for DMPC (0.25 mM and 1 mM), 324–325 nm for DOPC (0.25 mM and 1 mM), respectively (Fig. 6A–E). Similarly, we observed the unfolded HomB protein in 8 M urea demonstrated emission maximum at 340 nm and the folded proteins show emission maximum 323 nm for CHAPS (4×) and (10×), 324 nm for LDAO (4×) and (10×), 324–325 nm for TWEEN 20 (4×) and (10×), 324–323 nm for DMPC (0.25 mM and 1 mM), 324–323 nm for DOPC (0.25 mM and 1 mM), respectively (Fig. 6F–J). The emission spectra for LDAO, TWEEN 20, DMPC, and DOPC show increased fluorescence intensity for the folded proteins, suggesting that HomA and HomB structures are altered with the addition of these detergents and lipids. Furthermore, a blue-shift (hypsochromic shift) in the emission spectra observed during the refolding, induced by the addition of detergents and lipids, further confirms that both HomA and HomB restore the β-barrel topology in the presence of detergents and lipids.

### HomA and HomB exist in monomer and homodimer forms

**Figure 7:**
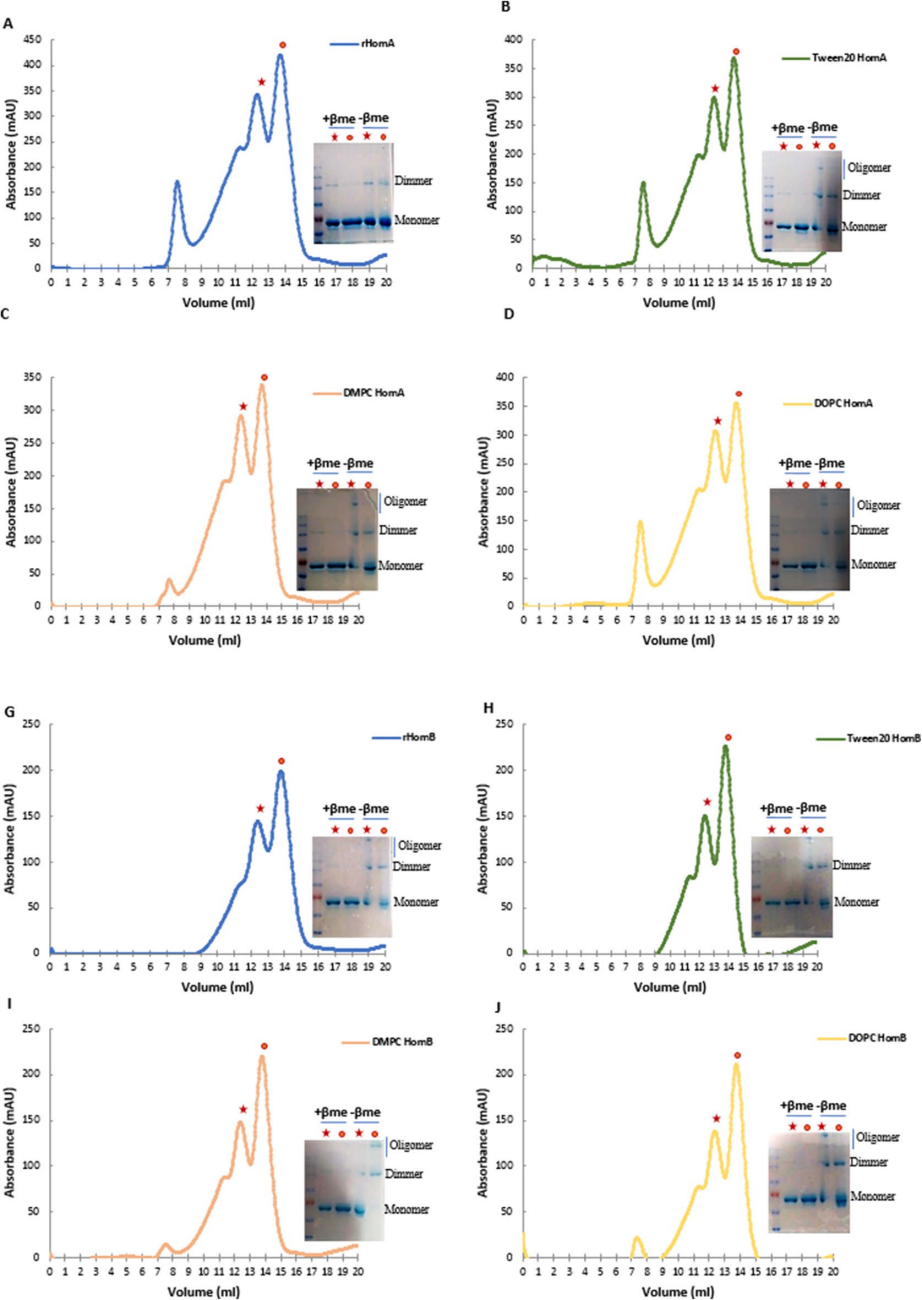
HomA and HomB exist in monomer and homodimer forms. (**A**) Chromatogram of size exclusion chromatography of HomA. (**B**–**D**) Size-exclusion chromatogram for HomA incubation with ×4 of CMC concentration of TWEEN 20, and 1 mM concentration of DMPC and DOPC lipids, respectively. (**E**) Chromatogram of size exclusion chromatography for HomB. (**F**–**H**) Size exclusion chromatogram of HomB incubated with ×4 of CMC concentration of TWEEN 20, and 1 mM concentration of DMPC and DOPC lipids, respectively. All the fractions were collected for monomer peak (red ball) and dimer (red star) for both HomA and HomB incubated with either detergent or lipid as mentioned and run onto SDS-PAGE with or without beta-mercaptoethanol (β-me).

To assess the oligomeric state of HomA and HomB, we performed size exclusion chromatography experiments. These investigations were carried out for HomA and HomB with the 10× concentration of CMC of detergents (LDAO, CHAPS, TWEEN 20) or 1 mM of lipids (DMPC, DOPC) in 50 mM Tris–Cl, pH 8.0, and 300 mM NaCl. The size exclusion chromatogram profile shows purified HomA and HomB was eluted in two major fractions which correspond to monomer and dimer forms (Fig. 7A). In the chromatogram for HomA, the first peak near 7.5 ml is of column void volume, the second hump like peak near 11 ml corresponds to the most likely trimer size of HomA. The third and fourth peaks at 12.5 and 13.5 ml correspond to dimer and monomer of HomA, respectively. The incubation of HomA with LDAO and CHAPS shows a major aggregation peak at column void volume but peaks that correspond to dimer and monomer still exist (Supplementary Fig. S4A,B). Incubation of HomA with TWEEN 20, DOPC and DMPC shows similar chromatograms. Simultaneously, fractions were collected analyzed on SDS-PAGE, and approximately 150 kDa and 75 kDa bands were observed which correspond to dimer and monomer size (Fig. 7B–D). Likewise, the HomB chromatogram shows prominent peaks for dimer and monomer at 12.5 and 13.5 ml, respectively (Fig. 7E). HomB was predominantly showing peaks for dimer and monomer, whereas the void volume peak was absent for HomB. Incubation of HomB with LDAO shows a peak at void volume, a shoulder peak near 9 ml and dimer, monomer peak at 12.5 and 13.5 ml, respectively (Supplementary Fig. S4C). However, incubation of HomB with CHAPS leads to aggregation, which shows a peak of void volume at 7.5 ml, although some fraction of dimer and monomer still exist (Supplementary Fig. S4D). DOPC, DMPC, and TWEEN 20 show similar chromatogram peaks with HomB, with little void volume peak in lipids (DOPC, DMPC) incubation (Fig. 7I,J). Like HomA, 150 kDa and 75 kDa bands were observed on SDS-PAGE for each collected fraction (Fig. 7F–H).

Incubation with detergents and lipids of HomA and HomB rearranges the secondary structure of proteins as observed in circular dichroism spectra (Fig. 4A–J). Here, in the size exclusion chromatography experiments, incubation with LDAO and CHAPS leads to larger size aggregation of both HomA and HomB proteins, whereas incubation with Tween 20 induces aggregation in HomA only. This suggests the rearrangement in the secondary structure of proteins due to the addition of detergents moieties leading to the formation of aggregates. However, the addition of lipids favours secondary structures that are similar to the wildtype proteins, and therefore do not show aggregation of proteins. Both proteins show the formation of the homodimer, as confirmed by SDS-PAGE analysis.

### Solution structure of HomA and HomB dimers

**Figure 8:**
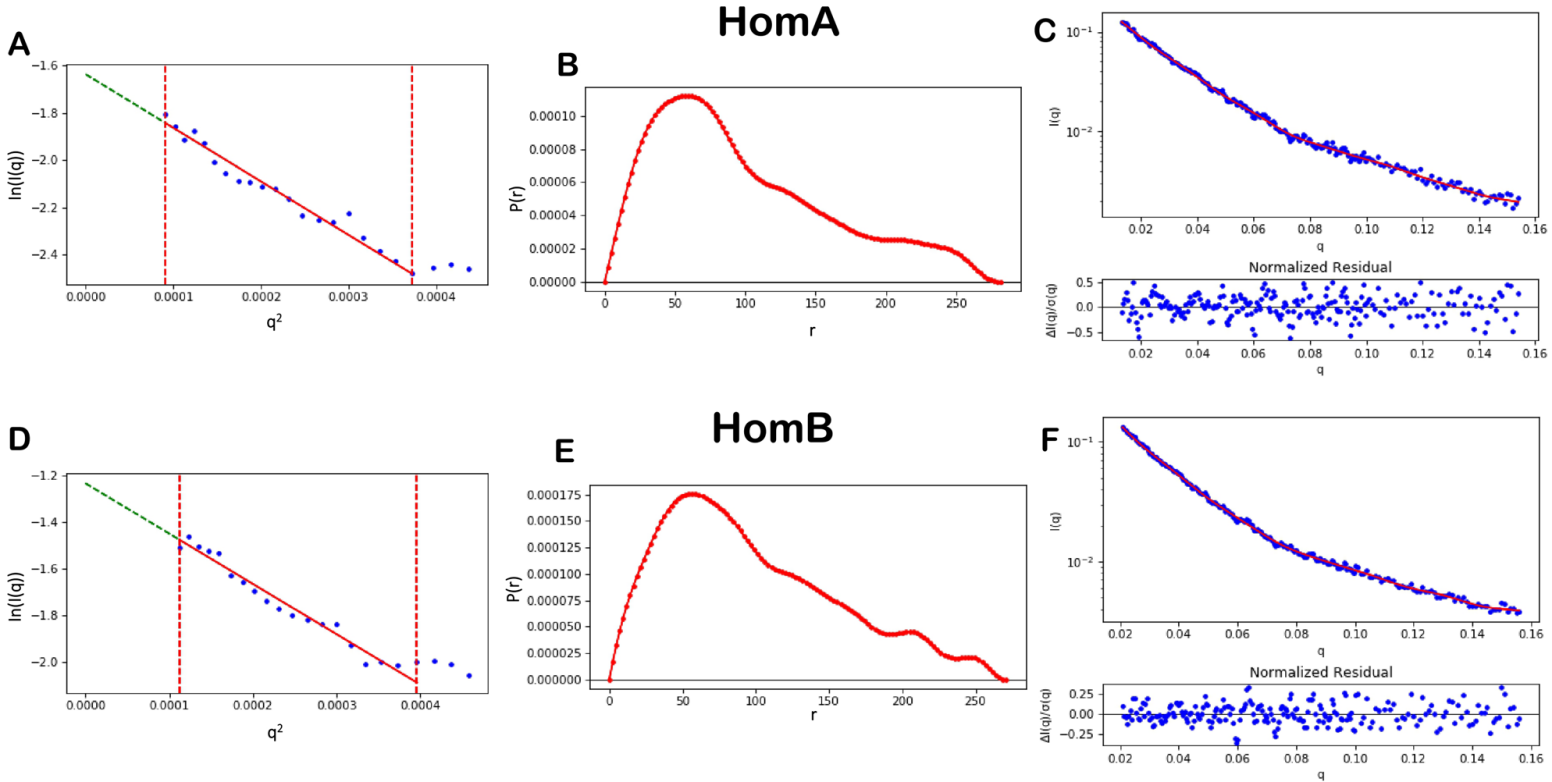
Guinier analysis of experimental SAXS data of HomA (**A**) and HomB (**D**). Pair distance distribution function [*P*(*r*)] for the experimental scattering data of HomA (**B**) and HomB (**E**). Indirect fourier transform (IFT) fit of experimental SAXS data of HomA (**C**; *χ*^2^ = 0.029) and HomB (**D**; *χ*^2^ = 0.0506) in red line and blue dots defines the experimental data.

**Figure 9:**
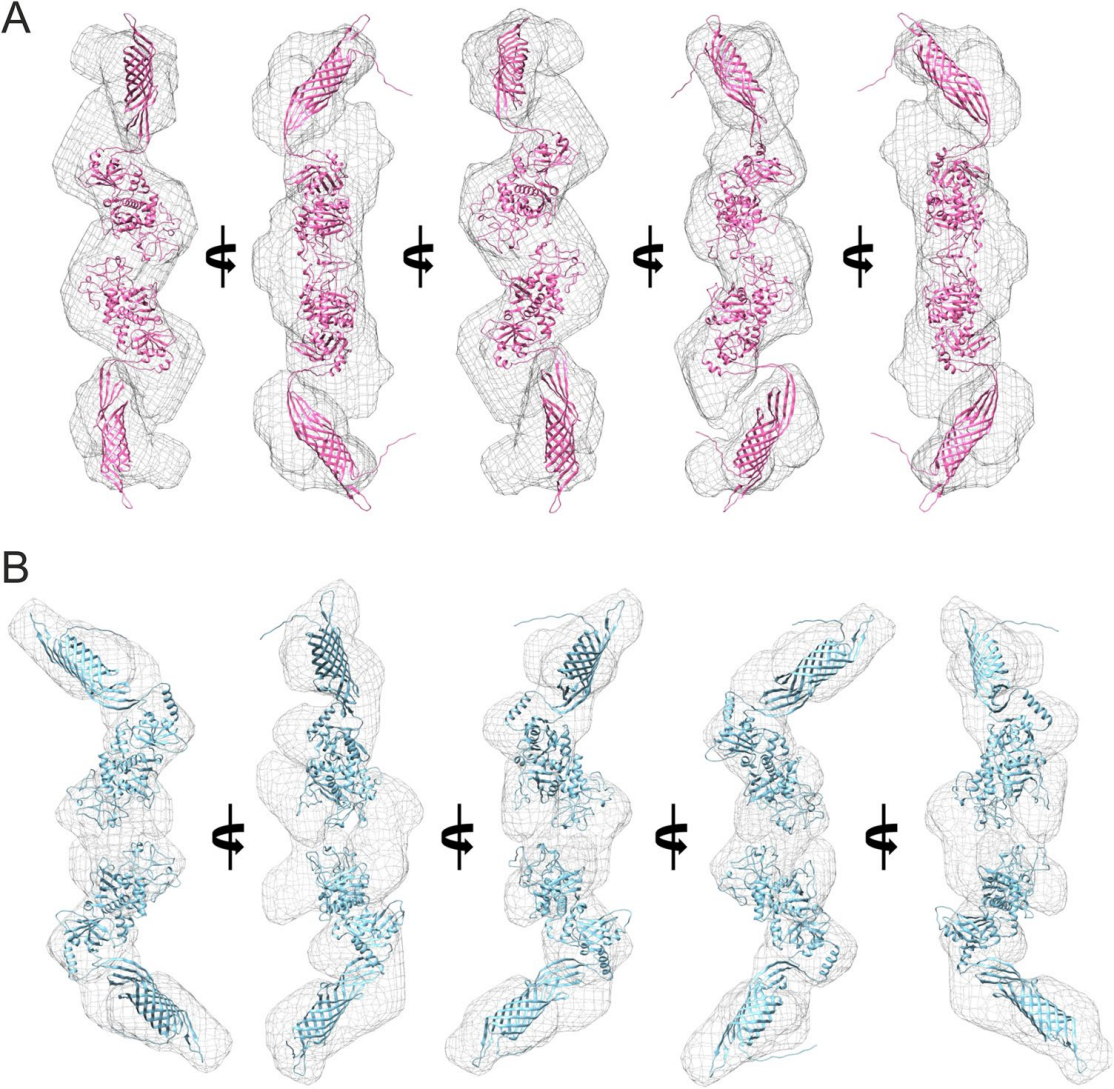
Ab initio shape model generated with DAMMIN (grey mesh; envelope at 15 Å resolution) and respective overlaid molecular structure of HomA (**A**) and HomB (**B**) in different orientations.

Small-angle solution scattering data on the aqueous solutions of HomA and HomB were acquired at the BL-18 beamline of the Indus-2 synchrotron source. For Guinier analysis, data with a q-range of 0.00955 to 0.0193 Å^−1^ for HomA and 0.0106 to 0.0199 Å^−1^ for HomB was analyzed. The Guinnier analysis showed a somewhat similar radius of gyration for HomA and HomB of around ~ 80 Å (Table 1 and Fig. 8). Indirect Fourier transformation (IFT) of the curves calculated using the GNOM program^31^ and the experimental curves fitted with SAXS data for HomA and HomB proteins were shown in (Table 1 and Fig. 8). The pair distance distribution function deduced maximum dimension (*D*max) for HomA and HomB was 280 and 271 Å^−1^ respectively. Both Guinier and pair distance distribution functions yielded similar *R*g values for both proteins, signifying the absence of aggregation or inter-particle interactions in samples (Table 1). Since the solution scattering data were collected on a relative scale of intensity, the molecular weight of both HomA and HomB from SAXS data was determined by concentration-independent methods^32,33^. The molecular weight estimated from the experimental SAXS profile (Table 1) indicates that both HomA and HomB are homodimers. Furthermore, AlphaFold 2 deduced the molecular structure of HomA and HomB fits exceptionally well against SAXS *ab-initio* shape models obtained with the DAMMIN module of ATSAS (Fig. 9).

**Table 1:** Properties deduced from solution scattering.

Sr. No.	Properties	HomA	HomB
**Guinier analysis**			
1	*I*_0_	0.1943 ± 0.0101	0.2907 ± 0.0188
2	*R*_g_ (Å)	82.4477 ± 4.8905	80.4194 ± 5.4411
3	*R*^2^	0.9665	0.9630
4	*q*_min_ (Å^−1^) (*q*_min_ × *R*_g_)	0.00955 (0.7874)	0.0106 (0.8524)
5	*q*_max_ (Å^−1^) (*q*_max_ × *R*_g_)	0.0193 (1.5912)	0.0199 (1.6003)
**Pair distance distribution function (IFT)**			
1	*I*_0_	0.1796 ± 0.0241	0.2865 ± 0.1626
2	*D*_max_ (Å)	282	271
3	*R*_g_ (Å)	82.6800	80.7100
4	*R*^2^	0.9665	0.9630
5	*q*_min_ (Å^−1^)	0.0132	0.0209
6	*q*_max_ (Å^−1^)	0.154	0.156
7	*X*^2^	0.0506	0.0166
**Molecular weight estimation (kDa)**			
8	SDS-PAGE	75 (monomer)/150 (dimer)	
9	Size exclusion chromatography	75 (monomer)/150 (dimer)	
10	SAXS	169.6^a^ (237.1)^b^	130.9^a^ (148.1)^b^

Molecular weight (MW) from SAXS data were determined from ^a^Porod’s volume^32^/^b^Porod’s volume calculated from truncated data (0.1 < *q*_max_ < 0.5 Å^−1^)^33^.

## Discussions

OMPs play a crucial role in several important physiological functions like ionic regulation, cell adhesion, host–pathogen interaction as well as pathogenesis, and structural information of these OMPs is vital for understanding their function and regulation. The present study provides several structural insights for OMPs of the Hom (*Helicobacter* outer membrane) family members of *H. pylori*, HomA and HomB. HomB is reported to be expressed at the *H. pylori* outer membrane and participates in bacterial adherence and inflammation. Furthermore, the dismissal of HomB causes significantly low production of IL-8. Moreover, it is also recognized by antibodies present in the human serum, which indicates that the surface-exposed regions of HomB likely interact with the host cell receptors^12^.

We performed bioinformatics analysis to predict the presence of a signal peptide (Fig. 1A), which ensures that these proteins are likely membrane proteins. Furthermore, we confirmed the presence of the C-terminal signature β-barrel sequence (Fig. 1B). Typically, β-barrel OMPs from Gram-negative bacteria, such as *E. coli* as well as others possess a C-terminal signature sequence, which makes these unique from other topological OMPs. This sequence helps OMPs to fold in a barrel shape, via the BAM complex^34^. Nevertheless, more recent works also reported another consensus having − 6 (glycine), − 3 (aromatic), − 2 (basic or large polar) and − 1 (aromatic) amino acids positions at C-terminal^35^. This consensus may slightly vary from Gram-negative species to species^36^. The *homB* and *homA* share 90% similarity, with only difference in the 300 bp central region. This difference does not make significant changes in the secondary structure and topology of both proteins.

Our in-silico analysis shows (Fig. 2A–H) membrane location of C-terminal conserved β-barrel domain for HomA and HomB where N-terminal β-strand is also a part of β-barrel (Supplementary Fig. S3A,B). I-TASSER also predicted a high C-score, a large size β-barrel model homologue with 22 β-strand bacterial plant-ferredoxin receptors FusA, for both HomA and HomB with slight variations (Supplementary Fig. S4A–F). However, trypsin digestion experiments suggest that the real structures for HomA and HomB are closer to the predicted models using AlphaFold 2 (Fig. 3A–D). Generally, bacteria display a great variety of outer membrane proteins that participates in nutrient transport, host signaling manipulation, adhesion, and virulence. Meuskens et al*.* reviewed different types of secretion systems of Gram-negative bacteria where these auto-transporters (ATs) have been categorized in 5 classes (Va-Ve), based on their structure and function characteristics. Additionally, they also describe another unique class of secretion system Vf. The Vf class of ATs contain a surface-exposed loop/globular domain inserted between the N and C-terminal regions^37^. These characteristics are unique structural features to the *H. pylori* Hop family outer membrane protein. For example, BabA protein shows a surface-exposed domain between the first and second β-strand of an 8-stranded β-barrel and contains no additional passenger at either terminus of the protein. Thus, the proposed passenger is an extended loop of the β-barrel domain and the β-barrel is smaller than that of any other ATs^24^. Similarly, Hom family members, HomA and HomB also likely to form disconnected small β-barrel where N-terminal β-strand (S1) is extended to a surface-exposed globular domain and connected back to C-terminal β-barrel with β-strand S19 in case of HomA and S20 in HomB (Fig. 3A,B). Although Vf class proteins are proposed to be a part of AT family, their structural topology is very different from the other types of ATs. Therefore, it is a subject of investigation whether these proteins do possess secretion mechanisms like other ATs or they possess some other unique feature for bacterial survival and pathogenesis. Heat modifiability is one of the peculiar features of β-barrel proteins where proteins show migration differences between boiled and unboiled samples applied to PAGE analysis^38^. We observed heat modifiability of HomA and HomB to some extent (Supplementary Fig. S1D,E), however, this was not as striking as some of the reports for other OMPs due to small β-barrel and large exposed loop. Normally, OMPs forming β-barrel structures are mostly rich in β-sheets. Many of the OMPs β-sheet structures are extensively studied with IR spectroscopy, which gives spectra containing a sum of peaks of the secondary structure content such, as β-sheet (parallel and antiparallel), α-helix, as well as coils/turns and this information lies under Amid I band of the spectra. HomA and HomB show a high β-sheet secondary structure content with parallel and anti-parallel β-sheets (Fig. 3E–H), which supports the in-silico secondary structure contents of being a β-barrel protein. Moreover, CD data strongly suggest that both HomA and HomB are rich in β-sheets that enables the formation of a β-barrel structure.

Renaturation of urea denatured HomA and HomB into a buffer containing detergents or lipids leads to changes in the secondary structure content of both proteins (Supplementary Table S3). We observed that the addition of detergents (LDAO and TWEEN 20) and lipids (DMPC and DOPC) favours either parallel or antiparallel β-sheets secondary structure (Fig. 4A–J) and possibly assists in folding or restoring these proteins to a native β-barrel structure. Conversely, we did not observe the restoration of the structure of HomA and HomB in the presence of CHAPS (Fig. 4B,G). In short, detergents (LDAO and TWEEN 20) and lipids (DMPC and DOPC) restore and favour the folded protein spectra, as CD experiments suggest that these lipids and detergents provide the most favourable environment for the refolding of urea denatured HomA, and CHAPS provides a least favoured environment for refolding of HomA and HomB proteins. Supplementary Table S4 summarises the folding ranking of the most to least favourable folding conditions for HomA and HomB in a few detergents and lipids, based on the circular dichroism spectra analysis.

Interestingly, HomA and HomB both are stable between pH range from 3 to 11 and remain in folded confirmations, as analyzed using Trp fluorescence experiments (Fig. 5A,B). A wide range of pH stability provides plasticity to proteins. Porin from *Yersinia pseudotuberculosis* was studied for its wide pH range (2.0 to 8.0) for its plasticity and structural rearrangement^39,40^. β-barrel proteins show a blue-shift and a few folds less intensity in Trp fluorescence spectra upon unfolding^41^. Trp spectra for HomA and HomB proteins show both 15 nm red-shifted fluorescence emission profiles along with a twofold loss in intensity upon unfolding in the presence of urea (Fig. 5C–E), similar to a typical characteristic of a transmembrane beta-barrel protein. Moreover, the addition of unfolded proteins in buffers containing detergents and lipids promotes the folding of the proteins (Fig. 6A–J). Incubation of unfolded HomA to buffer containing TWEEN 20 detergent shows blue shift but the intensity of spectra does not increase significantly which suggest it might not be the most favorable folding condition (Fig. 6C).

OMPs form β-barrel and can exist in multimeric forms. Arvind et al*.* characterized TrpC/D, a pore-forming OMP from *Treponema pallidum,* CD suggest that it forms β-barrel and exists in monomer and trimer forms^41^. Interestingly, we also observed that HomA and HomB proteins exist in two dominant forms, i.e. a monomer as well as a dimer. Earlier reports for OmpF from *E. coli* reassembled as a trimer are resistant to the denaturing action of sodium dodecyl sulfate (SDS) at room temperature^42,43^. The denatured monomer migrates faster than the dimer and trimer in a gel during SDS-PAGE. HomA and HomB also show resistance to denaturation by SDS when it was run on SDS-PAGE, and we observed both monomer and dimer bands. The addition of detergents leads to oligomerization of proteins, but dimer and monomer still exist, as evident from the SDS-PAGE analysis of fractions of size exclusion chromatography experiments performed with or without β-mercaptoethanol (Fig. 7A–H and Supplementary Fig. S3A–D).

The ab-initio structure of both HomA and HomB predicted from AlphaFold 2 has almost similar structural organization. Both proteins consist of two domains, the surface-exposed globular domain and a β-barrel domain with a hydrophobic patch suggesting its transmembrane nature (Fig. 3). Both domains were connected with a flexible neck, primarily consisting of loops. We believe this flexible neck provides the necessary degree of freedom for the surface-exposed globular domain against the membrane-anchored β-barrel domain to explore the best possible orientation for optimal ligand interaction. The size exclusion chromatography of both HomA and HomB shows the presence of dimer in the solution. The analysis of experimental SAXS data of both HomA and HomB showed the molecular weight of the scattering species very close to dimer (Table 1) supporting the presence of dimer species in solution. SDS-PAGE analysis of the dimeric fraction of HomA and HomB suggests the involvement of covalent interaction in dimerization (Fig. 7A–H and Supplementary Fig. S3A–D). Based on the SAXS data, the prepared dimer model of both proteins has their dimer interface in the loop region of the surface-exposed globular domain (Fig. 2). Interestingly, the four cysteine residues of HomA (245, 252, 393, 416) and all six cysteine residues (203, 215, 261, 268, 404, 427) of HomB are located in the loops of the globular domain (Supplementary Fig. S3). In such a surface-exposed oxidizing environment, these cysteine residues between homo (HomA_2_, HomB_2_) or hetero (HomA–HomB) globular domain could form disulfide bond/s to stabilize the oligomeric assembly. We believe that the oligomerization of HomA and HomB protein enables them to form stronger receptor-ligand interaction by harnessing the avidity of combined interaction between multiple receptors and ligands. Similar oligomeric association of receptor could be seen in Receptor tyrosine kinase signaling pathways like JAK-STAT signaling, insulin-GLUT4 signaling^44^, etc.

OMPs greatly help in the colonization of bacteria and persistent infection. For example, OMPs such as BabA, SabA, and HopC interact with Globo H, hexaglycosylceramide, Sialyl-Lewis, Collagen IV, Laminin, respectively^49^. Additionally, various β-barrel forming OMPs from *H. pylori*, HopE, and OMPLA help in the regulation and adaptation to the surrounding environment^50,51^. Thus, HomA and HomB dimers also play a crucial role in the biology of these proteins. Future experiments will aid in delineating how dimeric association is pivotal to the functions of these proteins in *H. pylori* pathogenesis.

Since these loop regions in β-barrel or the surface-exposed globular domain of OMP plays important role in oligomerization states^45^, and here also the surface-exposed globular domain likely contributes to dimer formation. Some of the hypothetical proteins (K74_10375, K747_09130, and K747_06625) and outer membrane protein is associated with biofilm formation, HomD, a member of Hom OMP family is associated with moderate biofilm former (58.3%) to hyper biofilm former (66.7%) *H. pylori* strains^46^. Recently, Stephanie et al*.* reported that *homB* expression is related to biofilm formation and regulated with pH and ArsRS dependent manner, and HomB helps *H. pylori* in hyper biofilm formation^47^. HomA and HomB both are stable at a wide range of pH from acidic to basic pH (Fig. 5A,B), it might be one of the reasons for biofilm formation and *H. pylori* survival in environmental pH stress conditions. The data presented herein gives structural insights that cysteine residues present on the surface-exposed domains (Fig. 3A,B) are expected to be involved in the homodimer formation of HomA and HomB. Such dimerizations assisted by HomA and HomB likely play an important role in cell–cell interactions, and thus might assist in biofilm formation. Biofilm formation is not only a crucial survival strategy but also aids in persistent and successful infection. Biofilm also protects bacteria from antibiotics and helps in evading immune response and *homB* is also associated with antibiotic resistance of *H pylori*^48^. C-terminal β-barrel of HomA and HomB forms pore-like structure topology (Fig. 3) and indicates porin channel-like properties, which might play an important role in the transport of biomolecules, including antibiotics. Investigation of HomA and HomB, especially their porin like functions, will not only provide a clue on their possible role in antibiotic resistance but also offer these proteins as potential therapeutic candidates. The further structural characterization will provide new insights into the molecular mechanism of *H. pylori* pathogenesis.

## Conclusions

*Helicobacter pylori* is the major cause of several stomach diseases. It interacts with gut epithelial cells and induces inflammation. Here, we explored the structural aspects of HomA and HomB which are reported for their prevalence in stomach diseases. Interestingly, in-silico studies demonstrated that HomA and HomB possess characteristics of β-barrel OMP, such as a signal peptide at the N-terminal and β-barrel signal at the C-terminal (Fig. 1A,B). HomA and HomB both have mixed secondary structure content, nevertheless, β-sheets are predominantly present in both proteins (Fig. 1C,F). Additionally, HomA and HomB localize in the outer membrane with 8 transmembrane antiparallel N + C-terminal β-strands connected by the large surface exposed globular domain (Fig. 2). Furthermore, the secondary structure was confirmed with ATR-FTIR, amid I spectra for HomA and HomB both contain mixed α helices and β-sheets spectra (Fig. 3E–H). CD spectra showed HomA and HomB both favors β-barrel structure and the addition of lipids and detergents to the denatured HomA and HomB rearranged proteins favoring antiparallel β-sheets (Fig. 4A–J). Interestingly, HomA and HomB were stable at a pH range from 3 to 11 (Fig. 5A,B). Additionally, both proteins start unfolding at 2 M of urea, the addition of denatured HomA and HomB to lipids, and detergents refold which was observed in the tryptophan fluorescence spectra (Fig. 6A–J). We performed size exclusion chromatography for HomA and HomB and observed peaks that correspond to dimer and monomer. Moreover, incubation of HomA and HomB with detergents shows oligomerization/aggregation of proteins, however, incubation with lipids shows peaks corresponding to dimer and monomer (Fig. 7A–H and Supplementary Fig. S5A–D). Furthermore, the solution structure analysis of HomA and HomB using small-angle X-ray scattering (SAXS) experiments confirmed that both HomA and HomB form homodimers (Fig. 9). OMPs are distinctive features of Gram-negative bacteria and play a crucial role in host–pathogen interaction, virulence and pathogenesis of bacteria. Our studies will help in a better understanding of *H. pylori* pathogenesis and host–pathogen interactions via OMPs.

## Methods

### Bioinformatics analysis of HomA and HomB

HomA (protein_id AAD06225.1) and HomB (protein_id AAD06437.1) amino acid sequences were used to identify the presence of signal peptides using the tool SignalP-5.0 (http://www.cbs.dtu.dk/services/SignalP/)^25^. We selected a Gram-negative signal peptide database for the prediction of a signal peptide in HomA and HomB. Secondary structure predictions for HomA and HomB were performed using NetSurfP-2.0 (http://www.cbs.dtu.dk/services/NetSurfP/)^27^. 3D structure predictions were done with AlphaFold2^28^ (https://deepmind.com/research/open-source/alphafold) and I-TASSER (https://zhanglab.ccmb.med.umich.edu/I-TASSER/)^29^.

### Cloning of *homA* and *homB*

*Helicobacter pylori* J99 (GenBank: AE001439.1) genomic DNA, a kind gift from Prof. Solnick, University of California, Davis, and Dr. Ashish Kumar Mukhopadhyay, NICED, Kolkata, was used as a template for amplifying *homA* (ORF jhp_0649) and *homB* (ORF jhp_0870) genes. The genes for HomA and HomB were PCR amplified from the genomic DNA of *H. pylori* strain J99, using *Pfu* DNA Polymerase with identical primer pair PK610 and PK652 (Supplementary Table S1) as the N-terminal and C-terminal of these proteins are conserved. Thermal cycles were programmed for 4 min as initial denaturation at 95 °C, followed by 30 cycles of 30 s at 92 °C for denaturation, 30 s at 55 °C as annealing temperature, 130 s at 72 °C for the extension, and a final extension at 72 °C for 10 min. Later, the PCR products were cloned into plasmid pBluescript at the *Sma*I site. Based on the restriction pattern, we identified unique clones for *homA* as well as *homB* and later confirmed by DNA sequencing. Subsequently, both the *homA* and *homB* genes were cloned into pET43 at *Nde*I and *Xho*I sites, respectively, to give pHomA and pHomB (Supplementary Fig. S1A). The cloning was performed in such a way that the recombinant proteins will express a C-terminal His-tag that will assist in purification. The inserts and vector DNA were ligated, followed by the transformation of ligated samples into DH5α cells. The presence of insert DNA in plasmid constructs was confirmed by colony PCR, further recombinant plasmids were isolated and then confirmed by DNA sequencing.

### Expression, purification and heat modifiability of recombinant HomA and HomB

Recombinant pHomA and pHomB clones were transformed into *E. coli* Rosetta cells. All recombinant strains were grown in a Luria–Bertani medium supplemented with (100 µg/ml) ampicillin. Cells were induced at an optical density at 600 nm of 0.6 by adding isopropyl-b-d-1-thiogalactopyranoside (IPTG) to a final concentration of 0.1 mM at 37 °C for 4 h on an orbital shaker at 220 rpm. Subsequently, the cells were harvested by centrifugation at 6000*g* for 15 min at 4 °C. The pellets were washed and resuspended in a buffer containing 30 ml of 25 mM Tris–Cl pH 7.5, 100 µg of lysozyme, and 500 µM of PMSF added and sonicated at 65-amp, 5 min pulse on and 5 min pulse off for 20 min for 2 cycles. After sonication, the pellet was recovered by centrifugation at 20,000*g* for 30 min at 4 °C. Subsequently, the recombinant HomA/HomB protein was purified on Ni-nitrilotriacetic acid affinity chromatography columns at 4 °C. Purification of recombinant HomA and HomB protein was confirmed on 8% SDS PAGE gel. Heat modifiability assay was done according to the method previously reported^45^, briefly, purified HomA and HomB with either detergent (LDAO, CHAPS, TWEEN20) with respective 4× CMC concentration and 1 mM lipids (DOPC and DMPC) added samples were boiled at 95 °C for 10 min and unboiled protein samples were run on 12% PAGE with 0.5% SDS at 150 V in cooling condition.

### Trypsin digestion

Recombinant purified proteins (0.5 mg/ml) were treated with 25 μg/ml of trypsin (SRL) for 1 h at 37 °C after which 5 mM phenyl-methylsulfonyl fluoride was added, and the samples were analyzed by SDS-PAGE and semi-native PAGE.

### Secondary structure analysis

Circular dichroism (CD) analysis was performed using a Jasco J-815 spectropolarimeter (Jasco, Easton, MD). Far-UV CD spectra were acquired at 25 °C in a 1-mm path-length cuvette, with a 1-nm bandwidth, and a scan rate of 20 nm/min. Spectra of each sample were acquired and the baseline was corrected by subtracting the spectral attributes of the buffer. The BeStSel tool (http://bestsel.elte.hu/) was utilized to assess the secondary structure contents of the proteins from their spectra^52^. Supplementary Fig. S8A,B illustrate curve fitting with minimal RMSD/NRMSD for all the CD data.

### Tryptophan fluorescence spectroscopy

Fluorescence spectra were obtained using the FluoroMax-4p spectrofluorometer from Horiba Jobin Yvon (model: FM100) with samples placed in a 1-mm path-length quartz cell at 25 °C temperature. The excitation wavelength was 295 nm, and the bandwidth of the excitation monochromator was 2 nm. The folding and denaturing buffers for HomA/HomB were 50 mM Tris–Cl, pH 8.0, 300 mM NaCl, 1–8 M urea. Tryptophan emission spectra were recorded between 305 and 400 nm. Background spectra without HomA/HomB were subtracted to obtain the final emission curves.

### Size exclusion chromatography

For determination of the oligomeric state of HomA and HomB, 1 mg of protein was loaded onto a Superose 12 size exclusion column (GE Healthcare) equilibrated with 50 mM Tris–Cl, pH 8.0, 300 mM NaCl, and lipid and detergent as indicated, at 4 °C. Protein was eluted at a flow rate of 0.5 ml/min. The standard molecular weight markers used were 200 kDa (β amylase), 150 kDa (alcohol dehydrogenase), 66 kDa (albumin), 29 kDa (carbonic anhydrase), and 12 kDa (cytochrome C) (Supplementary Fig. S3A,B). The void volume is determined by blue dextran of MW ~ 2000 kDa.

### ATR-FTIR

Polarized ATR infrared spectra were recorded on a Tensor 27 (Bruker) FTIR spectrometer at a resolution of 2 cm^−1^, with parallel and perpendicular polarization of the incident beam. 80–100 µg of purified protein was used in a buffer containing 50 mM Tris–Cl, pH 8.0, 300 mM NaCl. Quantitative analysis of the amide I band contour was done using curve fitting, second derivative methods. The spectral range of 1600–1700 cm^−1^ was decomposed using Origin 8.0 software. Buffer spectra without protein were subtracted to obtain the final spectra.

### SAXS data acquisition, processing, analysis and shape restoration

SAXS measurements were carried out at the SAXS beamline (BL-18) of the Indus-2 synchrotron (2.5 GeV, 300 mA) source, Indore, India. The X-ray beam of wavelength 0.7749015 Å (16 keV) was used. HomA and HomB protein samples (~ 2.5 mg/ml) in buffer containing 20 mM Tris–Cl pH 8, 200 mM NaCl, and 5 mM BME were placed inside a “Kapton window cell”, with 4 mm path length for SAXS data collection. The data were collected in a *q* (4πsin*ϴ*/*λ*, where *ϴ* is the scattering angle) range from 0.0096 to 0.361 Å^−1^ for both HomA and HomB using MAR345 imaging plate detector with the sample to detector distance of 3.24 m. The data were corrected for both solvent and background, and the intensities were radially averaged on a relative scale. For both data-set, the data in the *q*-range 0.095 to 0.154 Å^−1^ for HomA and 0.010 to 0.156 Å^−1^ for HomB were analyzed and modeled mostly by using software tools available in the ATSAS suite^53^. The radius of gyration (*R*g) and absolute intensity *I*(0) (at *q* = 0) values were estimated from pair distance distribution [*P*(*r*)] were calculated by GNOM^31^ of ATSAS suite^54^. The molecular weight of the HomA and HomB were estimated in a concentration-independent manner from Porod volume^32,33^ implemented in RAW software^55^, as SAXS data were on an arbitrary relative scale. Ab-initio bead model for both proteins was generated by the DAMMIN module of the ATSAS package. Simultaneously, the protein structures of HomA and HomB were predicted from AlphaFold 2 online server^28,56^. The dimeric assembly for both HomA and HomB was manually designed based on the SAXS bead model by using PyMOL. Data have been deposited in the Small Angle Scattering Biological Databank (www.sasbdb.org; ID: HomA; SASDMK7 and HomB; SASDML7). Structural figures were prepared using and UCSF Chimera.

## Supplementary Information

Supplementary Information.
