# Supplementary material for: Biophysical characterization of the homodimers of HomA and HomB, outer membrane proteins of Helicobacter pylori
Source: Sci Rep. 2021 Dec 28;11:24471. doi: 10.1038/s41598-021-04039-4 (PMC8714817; doi:10.1038/s41598-021-04039-4)
Supplement: Supplementary file 1 — Supplementary Information. [file 41598_2021_4039_MOESM1_ESM.pdf]

Electronic Supplementary Information

Biophysical characterization of the homodimers of HomA and HomB, outer membrane proteins of *Helicobacter pylori*

Anubhav Tamrakar[1], Rahul Singh[2], Amit Kumar[1], Ravindra D. Makde[2], Ashish[3], and Prashant Kodgire[1]*

[1] Discipline of Biosciences and Biomedical Engineering, Indian Institute of Technology Indore, Indore - 453 552, Madhya Pradesh, India

[2] High Pressure and Synchrotron Radiation Physics Division, Bhabha Atomic Research Center, Trombay, Mumbai, India.

[3] Protein Science and Engineering Division, CSIR-Institute of Microbial Technology, Chandigarh, India.

* Correspondence should be addressed to Dr. Prashant Kodgire

Email : pkodgire@iiti.ac.in

Phone : 91-731 6603355

Table S1. Primers used for cloning of *homA and homB*

S No	Primer	Sequence (5'-3')[a]	Restriction site[b]
1	PK610F	GGC**CATatg**agaaaactattcatcccacttttatta	*Nde*I
2	PK652R	ATGC**CTCGAG**aaacacccacccgtaattg	*Xho*I

[a] Nucleotides in the small case are complementary to the genome sequence.

[b] Underlined (bold case) in the corresponding sequence.

Table S2. Secondary structure content analysis based on selective region (1600-1700 cm^{-1}) FTIR second derivative spectrum of HomA and HomB

S No	Secondary structure content	Peak position (cm^{-1})	
		HomA	HomB
1	Sidechain	1612	1612
2	Anti-parallel β-sheets	1624	1625
3	Parallel β-sheets	1634	1634
4	Disordered	1643	1643
5	α-Helix	1654	1653
6	β-sheets+Turn	1664, 1677	1664, 1678
7	Anti-parallel β-sheets (weak)	1685, 1695	1685, 1697

Table S3. Secondary structure analysis using circular dichroism spectra

S No	Sample	α-Helix %		Anti-parallel β-sheets %			Parallel β-sheets %	Turn %	Other %	NRMSD
		Regular	Distorted	Left twisted	Relaxed	Right twisted				
1	HomA	10.5	0	22.1	0	3.5	13.5	11..5	38.4	0.09
2	HomA CHAPS (4X)	8	0	12.7	0	0	0	52.9	26.4	0.11
3	HomA CHAPS (10X)	11.0	0	17.7	0	0	0	50	21.3	0.14
4	HomA TWEEN 20 (4X)	12.0	0	6.4	0	13.2	38.7	0	29.7	0.11
5	HomA TWEEN 20 (10X)	11.0	0	4.1	0	15.4	39.3	0	30.2	0.10
6	HomA LDAO (4X)	0	0	1.1	0	7.4	33.9	12.5	45.1	0.03
7	HomA LDAO (10X)	0	3.3	0	0	10.7	41.7	1.8	42.6	0.04
8	HomA DMPC (0.250mM)	14.1	0	10.4	0	9.6	38.2	0	27.7	0.10
9	HomA DMPC (0.500mM)	14.7	0	15.1	0	7.4	28.4	4.5	29.9	0.09
10	HomA DMPC (1mM)	16.2	0	14.9	0	7.9	25.2	4.7	31.2	0.10
11	rHomA DOPC (0.250mM)	11.6	0	13.9	0	7.7	32.3	3.2	31.3	0.09
12	rHomA DOPC (0.500mM)	15.8	0	15.5	0	6.7	27.3	4.9	29.8	0.10
13	HomA DOPC (1mM)	16.0	0	22.7	0	3.1	17.3	10.6	30.2	0.10
14	HomB	12.0	8.0	0	1.6	10.0	14.4	14.4	39.9	0.02
15	HomB CHAPS (4X)	0	2.7	11.8	3.3	13.2	3.8	23.2	41.9	0.05
16	HomB CHAPS (10X)	9.1	0	15.9	0	0	0	47.7	27.3	0.12
17	HomB TWEEN 20 (4X)	9.7	7.3	0	5.5	11.3	13.9	13.4	38.9	0.02
18	HomB TWEEN 20 (10X)	9.7	7.3	0	5.5	11.3	13.9	0	38.9	0.02
19	HomB LDAO (4X)	8.2	9.0	0	7.1	14.8	6.2	14.6	40.1	0.02
20	HomB LDAO (10X)	13.8	10.0	0	2.2	11.5	9.3	14.1	39.1	0.02
21	HomB DMPC (0.250mM)	5.5	7.4	0.9	10.8	17.6	4.1	13.7	39.9	0.02
22	HomB DMPC (0.500mM)	15.4	10.6	0	0	9.5	10.3	13.4	40.8	0.02
23	HomB DMPC (1mM)	5.8	6.9	0.8	10.4	17.5	5.2	13.4	40.0	0.02
24	HomB DOPC (0.250mM)	6.8	6.8	0	9.2	15.6	8.5	13.5	39.6	0.02
25	HomB DOPC (0.500mM)	5.1	7.0	1.1	11.1	16.6	6.0	13.7	39.5	0.02
26	HomB DOPC (1mM)	6.0	6.6	0	10.9	16.6	7.5	13.4	39.0	0.02

Table S4. Most to least favourable folding conditions for HomA and HomB in detergents and lipids based on the circular dichroism spectra analysis

**Folding ranking**	**Detergent and lipids**	
	**HomA**	**HomB**
High	LDAO	LDAO; DOPC and DMPC; TWEEN20
Moderate	DOPC and DMPC; TWEEN20	
Low	CHAPS	CHAPS

Figure S1

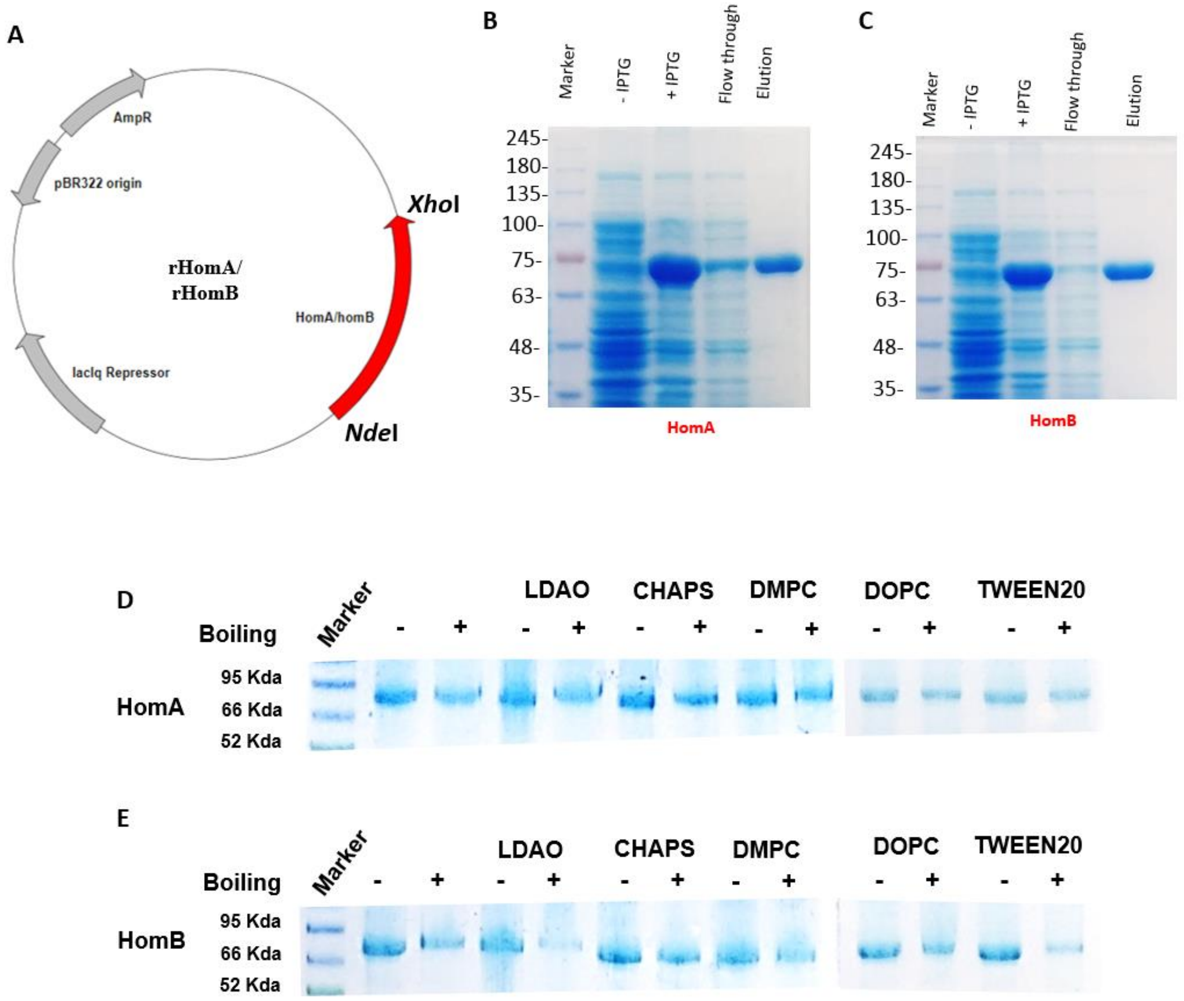

Figure S1. Cloning, expression, purification and heat modifiability of HomA and HomB. A. Cloning strategy of HomA and HomB **B-C**. SDS-PAGE of expression and purification gels of HomA and HomB respectively. For both proteins, SDS-PAGE lane details are: Lane 1, protein marker. Lane 2, uninduced supernatant. Lane 3, induced supernatant. Lane 4, flow-through (purification fraction). Lane 5, elution (purification fraction). **D-E.** Heat modifiability PAGE of HomA (**D**) and HomB (**E**).

Figure S2

```
AAD06225.1      MRKLFIPLLLFSALEANEKNGFFIEAGFETGLLEGTQTQEKRHTTTKNTYATYNYLPTDT    60
AAD06437.1      MRKLFIPLLLFSALEANEKNGFFIEAGFETGLLEGTQTQEKRHTTTKNTYATYNYLPTDT    60
                ************************************************************

AAD06225.1      ILKRAANLFTNAEAISKLKFSSLSPVRVLYMYNGQLTIENFLPYNLNNVKLSFTDAQGNV    120
AAD06437.1      ILKRAANLFTNAEAISKLKFSSLSPVRVLYMYNGQLTIENFLPYNLNNVKLSFTDAQGNT    120
                ***********************************************************.

AAD06225.1      IDLGVIETIPKHSKIVLPGEAFDSL-----KIDPYTLFLPKIEATSTSISDANTQRVFET    175
AAD06437.1      IDLGVIETIPKHSKIVLPGEAFDSLKEAFDKIDPYTLFLPKFEATSTSISDTNTQRVFET    180
                *************************     ***********:*********:********

AAD06225.1      LNKIKTNLVVNYRNENKF-----------KDHENHWEAFTPQTAEEFTNLMLNMIAVLDS    224
AAD06437.1      LNNIKTNLIMKYSNENPNNFNTCPYNNNGNTKNDCWQNFTPQTAEEFTNLMLNMIAVLDS    240
                **:*****:::* ***             : ::: *: **********************

AAD06225.1      QSWGDAILNAPFEFTNSPTDCDNDPSKCVNPGTNGLVNSKVDQKYVLNKQDIVNKFKNKA    284
AAD06437.1      QSWGDAILNAPFEFTNSSTDCDSDPSKCVNPGVNGRVDTKVDQQYILNKQGIINNFRKKI    300
                ***************** ****.*********.** *::****:*:****.*:*:*::*

AAD06225.1      DLDVIVLKDSGVVGLGSDITPSNNDDGKHYGQLGVVASALDPKKLFGDNLKTINLEDLRT    344
AAD06437.1      EIDAVVLKNSGVVGLANGY-----GNDGEYGTLGVEAYALDPKKLFGNDLKTINLEDLRT    355
                ::*.:***:******...      .:. .** *** * *********::***********

AAD06225.1      ILHEFSHTKGYGHNGNMTYQRVPVTKDGQVEKDSNGKPKDSDGLPYNVCSLYGGSNQPAF    404
AAD06437.1      ILHEFSHTKGYGHNGNMTYQRVPVTKDGQVEKDSNGKPKDSDGLPYNVCSLYGGSNQPAF    415
                ************************************************************

AAD06225.1      PSNYPNSIYHNCADVPAGFLGVTAAVWQQLINQNALPINYANLGSQTNYNLNASLNTQDL    464
AAD06437.1      PSNYPNSIYHNCADVPAGFLGVTAAVWQQLINQNALPINYANLGSQTNYNLNASLNTQDL    475
                ************************************************************

AAD06225.1      ANSMLSTIQKTFVTSSVTNHHFSNASQSFRSPILGVNAKIGYQNYFNDFIGLAYYGIIKY    524
AAD06437.1      ANSMLSTIQKTFVTSSVTNHHFSNASQSFRSPILGVNAKIGYQNYFNDFIGLAYYGIIKY    535
                ************************************************************

AAD06225.1      NYAKAVNQKVQQLSYGGGIDLLLDFITTYSNKNSPTGIQTKRNFSSSFGIFGGLRGLYNS    584
AAD06437.1      NYAKAVNQKVQQLSYGGGIDLLLDFITTYSNKNSPTGIQTKRNFSSSFGIFGGLRGLYNS    595
                ************************************************************

AAD06225.1      YYVLNKVKGSGNLDVATGLNYRYKHSKYSVGISIPLIQRKASVVSSGGDYTNSFVFNEGA    644
AAD06437.1      YYVLNKVKGSGNLDVATGLNYRYKHSKYSVGISIPLIQRKASVVSSGGDYTNSFVFNEGA    655
                ************************************************************

AAD06225.1      SHFKVFFNYGWVF    657
AAD06437.1      SHFKVFFNYGWVF    668
                *************
```

Figure S2 Protein Sequence alignment between HomA (AAD06225.1) and HomB (AAD06437.1). Sequence alignment was performed by Clustal Omega. Stars are showing identity. The double dot indicates strongly similar properties and the single dot indicates weakly similar properties. Gaps are showing not identical and not similarity in the alignment.

Figure S3.

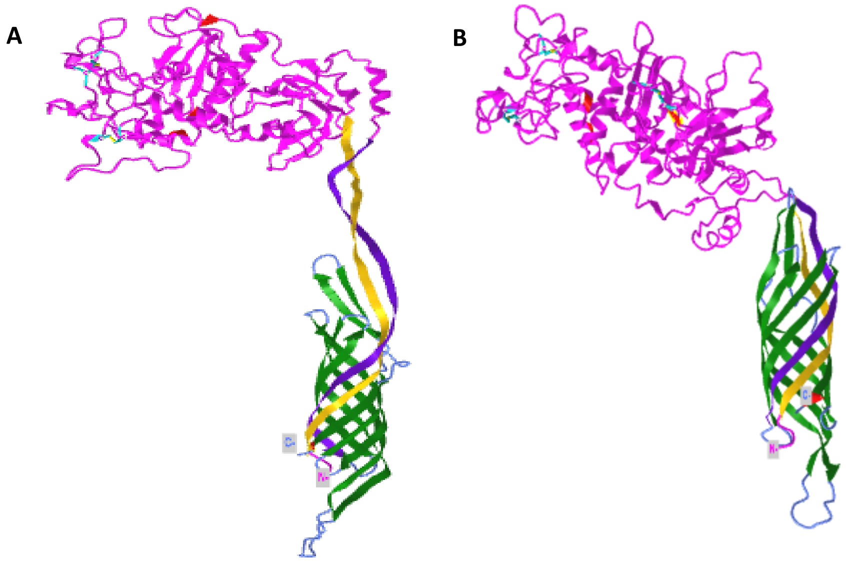

Figure S3. Alphafol2 3D structure representation of HomA and HomB. A-B. **A** HomA and **B** HomB, forms small β-barrel, N-terminal S1 β-strand (yellow) is part of β-barrel which extend to form surface-exposed large globular domain and S19 (HomA) and S20 (HomB) is β-strand (violet) joined back β-barrel. Cystine residues (cyan) in the surface-exposed globular domain of HomA are at 245, 252, 393, and of HomB at 203, 215, 262, 268, 404 and 427, respectively. Tryptophan residues (red) are present in the surface-exposed globular domain as well as β-barrel.

Figure S4

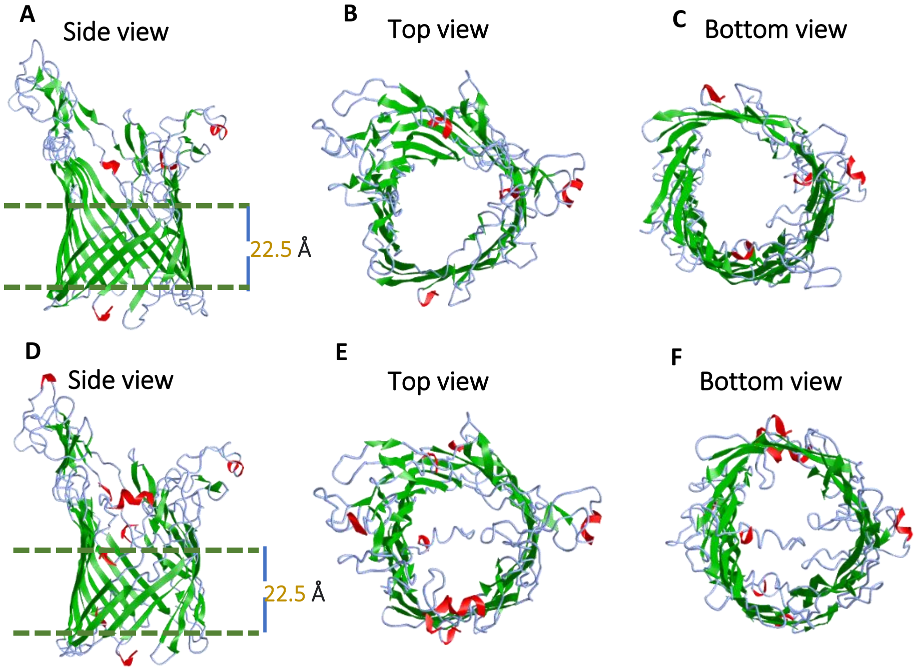

Figure S4. I-TASSER 3D structure prediction of HomA and HomB. A-L. 3D structure predictions of HomA and HomB (without signal peptide) using I-TASSER (https://zhanglab.ccmb.med.umich.edu/I-TASSER/)[27], side, top, and bottom view for HomA from left to right (**A-C)**; side, top and bottom view for HomB from left to right (**D-F).**

Figure S5. A

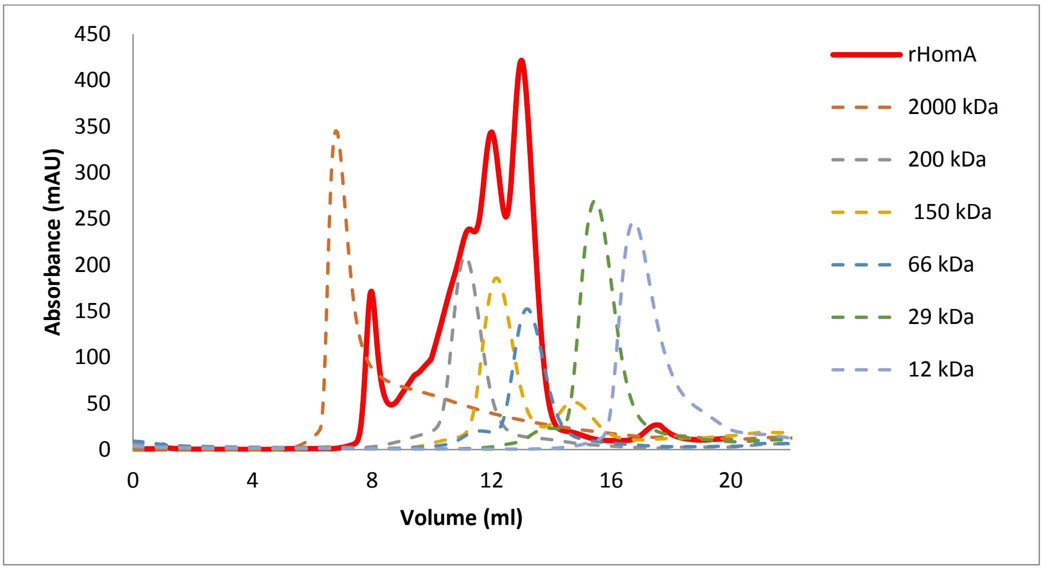

Figure S5. B

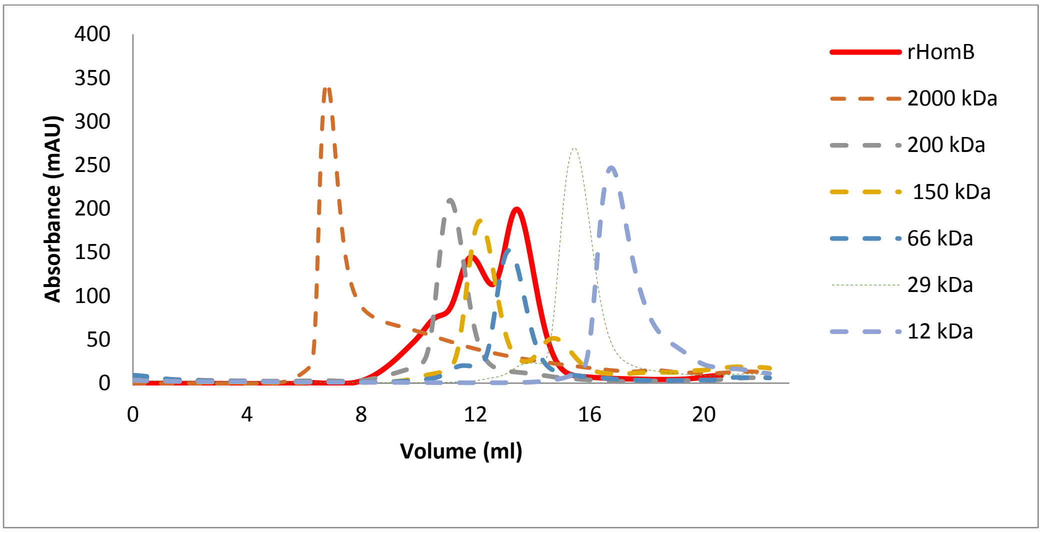

Figure S5 A-B. Gel filtration profile of HomA (A) and HomB (B) on Superose 12 column in buffer 50 mM Tris-Cl pH 8.0, 300 mM NaCl. The standard molecular weight markers used were 200 kDa (β amylase), 150 kDa (alcohol dehydrogenase), 66 kDa (albumin), 29 kDa (carbonic anhydrase), and 12 kDa (cytochrome C). The void volume is determined by blue dextran (red colour profile) of MW~ 2000 kDa. Molecular weight for HomA and HomB is estimated to be about 75-70 kDa approx for monomer peaks respectively and 150 kDa approx which is equivalent to its dimer.

Figure S6. A

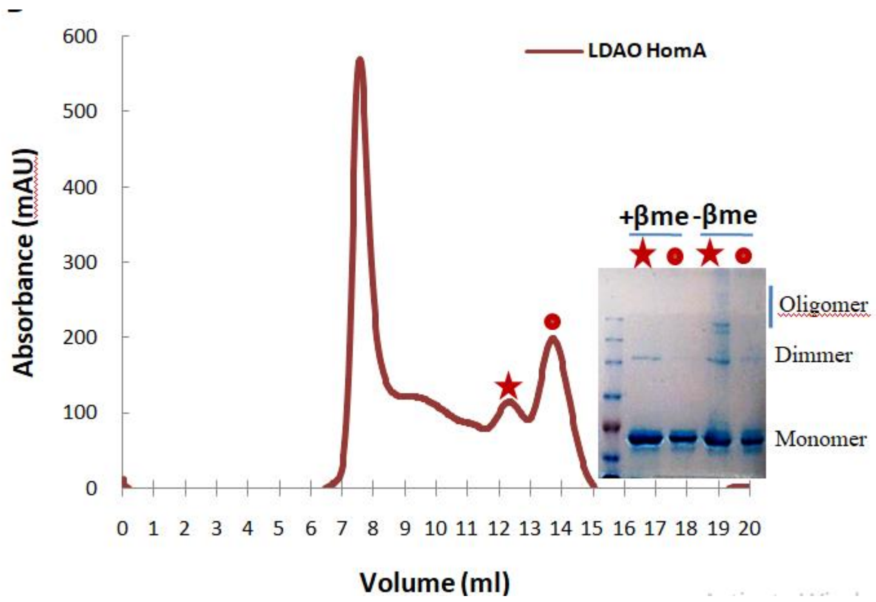

Figure S6. B

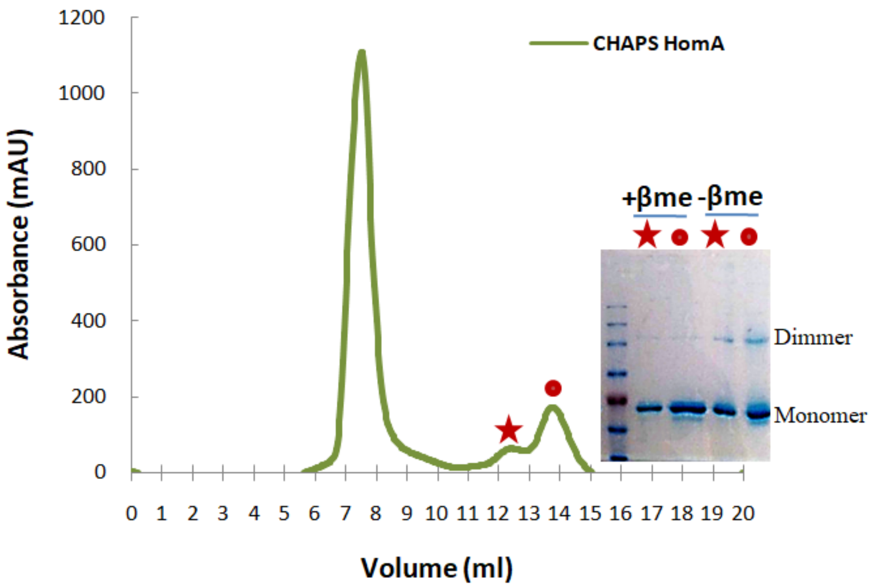

Figure S6. C

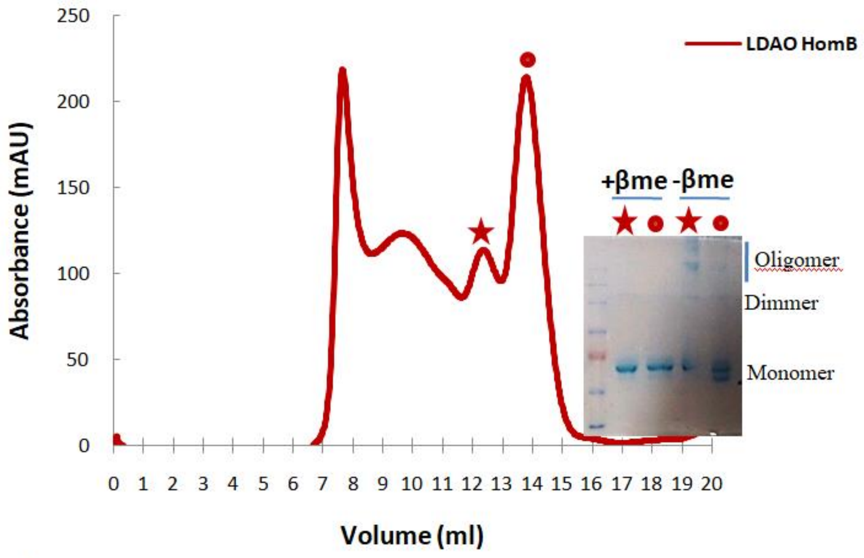

Figure S6. D

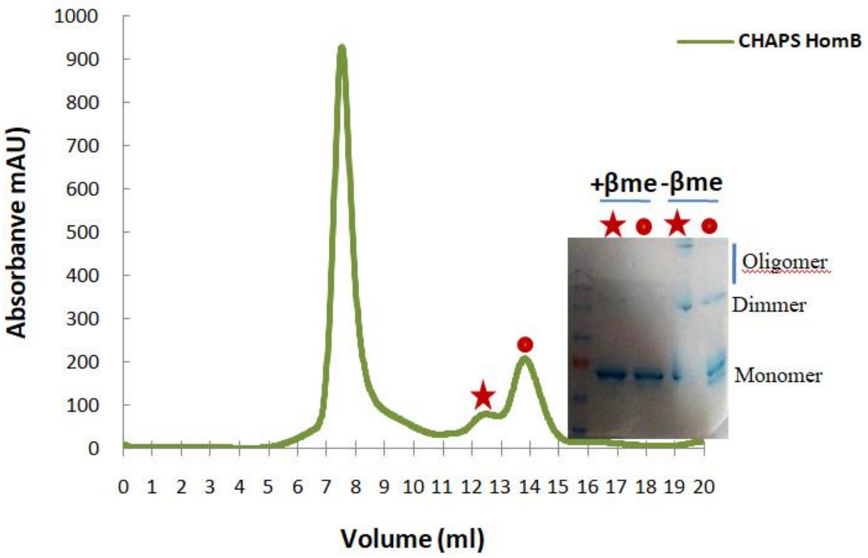

Figure S6. A, B. Size-exclusion chromatogram for HomA incubation with 10X of CMC concentration of LDAO, CHAPS, and **C, D.** Size-exclusion chromatogram for HomA incubation with 10X of CMC concentration of LDAO, CHAPS. All the fractions were collected for monomer peak (red ball) and dimer (red star) for both HomA and HomB incubated with either detergent or lipid as mentioned, and run onto SDS-PAGE with or without beta-mercaptoethanol (β-me).

Figure S7.

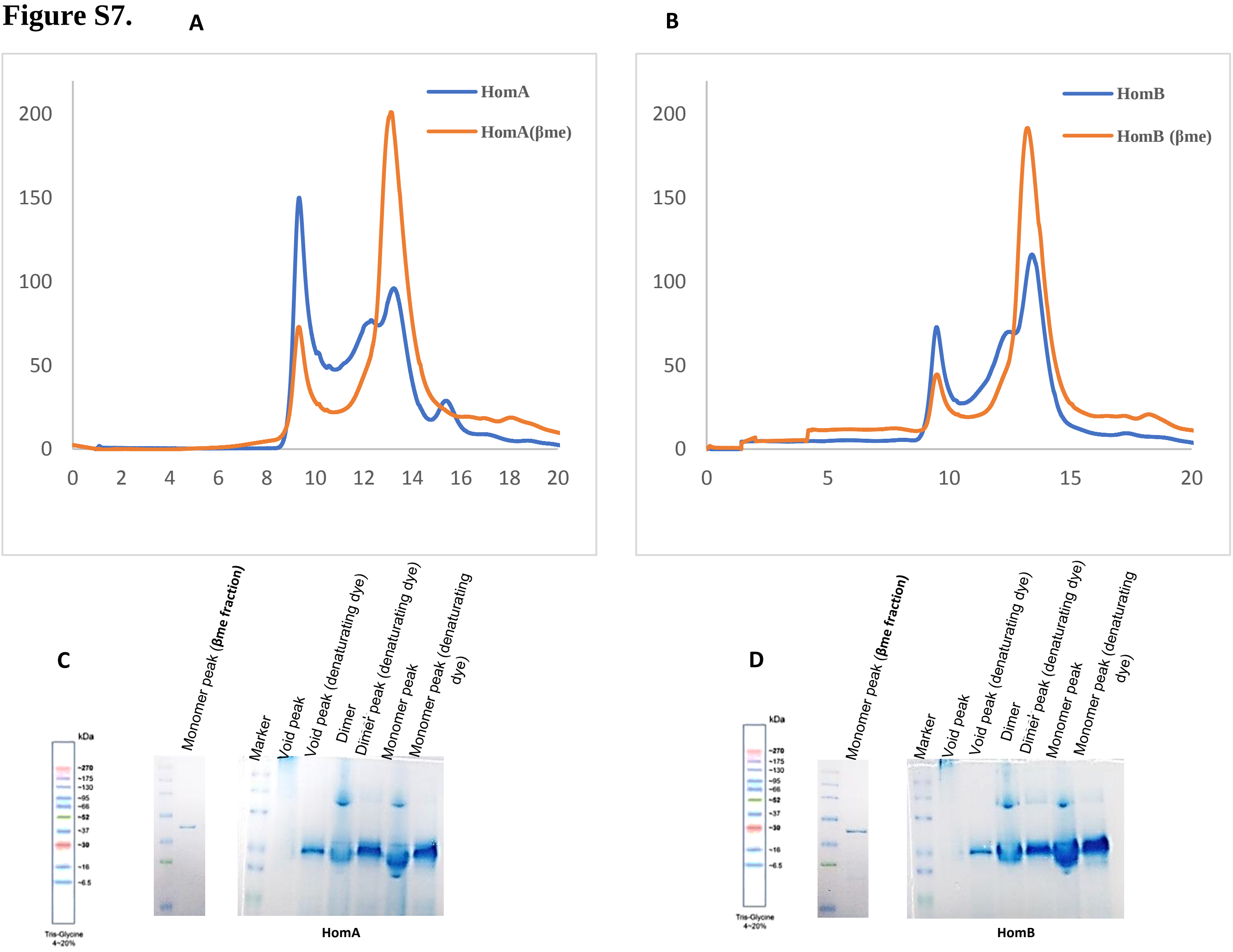

Figure S7. A-B. Size-exclusion chromatogram for HomA and HomB in buffer with and with or without beta-mercaptoethanol (β-me). Monomer peak with dimer shoulder observed in protein sample without beta-mercaptoethanol (β-me) buffer and dimer shoulder peak was not observed in protein sample with beta-mercaptoethanol (β-me, 5mM) buffer. **C-D.** size exclusion chromatography fractions collected and run onto the SDS-PAGE.

Figure S8.

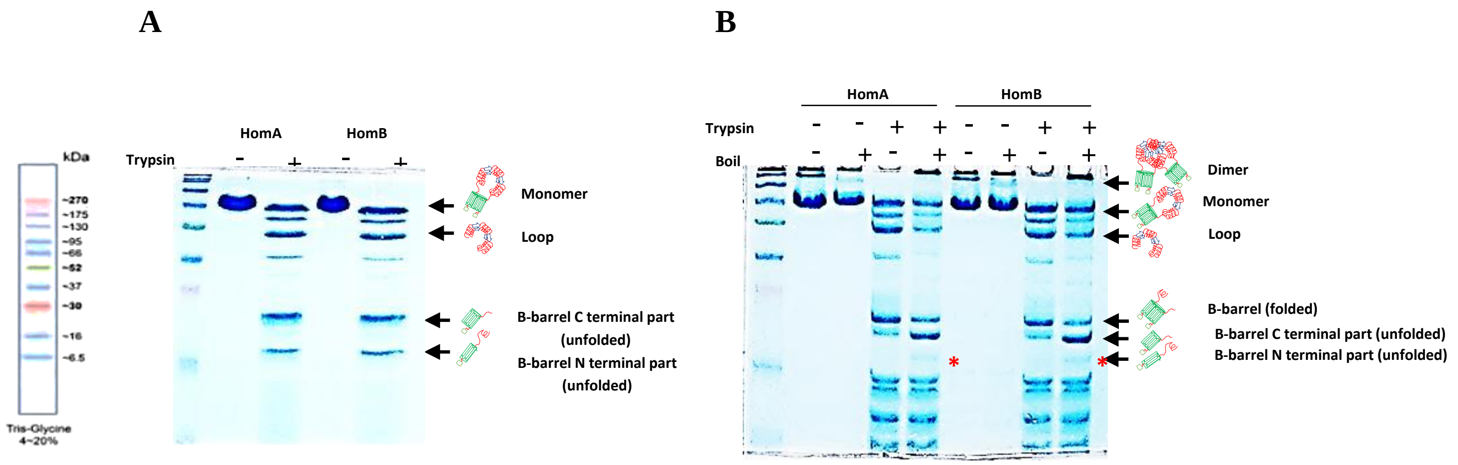

Figure S8. Trypsin digestion analysis of HomA and HomB. A, trypsin digested proteins run onto denaturing SDS-PAGE. **B**, same protein sample run onto the semi-native gel with and without boiling. (**Full gel image for Fig. 3B** and **C).**
